# RSV Hospital Admissions During the First 2 Seasons Among Children With Chronic Medical Conditions

**DOI:** 10.1001/jamanetworkopen.2025.19410

**Published:** 2025-07-08

**Authors:** Marina Viñeta Paramo, Allison W. Watts, Jeffrey N. Bone, Manish Sadarangani, Julie A. Bettinger, Claire Seaton, Alfonso Solimano, Matthew O. Wiens, Danuta M. Skowronski, Hind Sbihi, Pascal M. Lavoie

**Affiliations:** 1 Department of Pediatrics, University of British Columbia, Vancouver, British Columbia, Canada; 2 Women+ and Children’s Health, Department of Obstetrics and Gynecology, University of British Columbia, Vancouver, British Columbia, Canada; 3 British Columbia Children’s Hospital Research Institute, Vancouver, British Columbia, Canada; 4 Vaccine Evaluation Center, British Columbia Children’s Hospital Research Institute, Vancouver, British Columbia, Canada; 5 Department of Anesthesiology, Pharmacology & Therapeutics, Faculty of Medicine, University of British Columbia, Vancouver, British Columbia, Canada; 6 Immunization Programs and Vaccine Preventable Diseases Service, British Columbia Centre for Disease Control, Vancouver, British Columbia, Canada; 7 Data and Analytics Services, British Columbia Centre for Disease Control, Vancouver, British Columbia, Canada; 8 School of Population and Public Health, University of British Columbia, Vancouver, British Columbia, Canada

## Abstract

**Importance:**

National Immunization Technical Advisory Groups recommend long-acting monoclonal antibody prophylaxis for the prevention of respiratory syncytial virus (RSV) disease for children at high risk in the first season, regardless of RSV vaccination during pregnancy, and for those who remain at increased risk in the second season.

**Objective:**

This study assessed which groups of children with chronic medical conditions (CMCs) are at higher risk of RSV hospitalization during their first and second RSV seasons.

**Design, Setting, and Participants:**

This retrospective, population-based, season-stratified cohort analysis was conducted among children who were born between April 1, 2013, and March 31, 2023, in British Columbia, Canada (population of 5.7 million in 2024), and were enrolled in the provincial medical service plan and followed up until the day before their third RSV season or April 1, 2024, whichever occurred first.

**Exposure:**

Any CMC diagnosed in the first 2 years of life.

**Main Outcomes and Measures:**

Respiratory syncytial virus–related hospitalizations.

**Results:**

The final cohort consisted of 431 937 children (32 959 [7.6%] born at <37 weeks’ gestation; 222 207 boys [51.4%]) followed up for a median of 728 days (IQR, 642-729 days), including 25 452 children (5.9%) diagnosed with at least 1 of 1116 distinct CMCs. In total, 4567 children (1.1%) experienced a combined total of 4592 RSV hospitalizations, combining data from the first and second RSV seasons. In the first RSV season, the RSV hospitalization rate per 1000 person-years for children with CMCs was 15.9 (95% CI, 14.2-17.6) and for children without CMCs was 8.0 (95% CI, 7.7-8.3). In the second RSV season, the RSV hospitalization rate per 1000 person-years for children with CMCs was 7.8 (95% CI, 6.7-8.8) and for children without CMCs was 2.2 (95% CI, 2.1-2.3). Children with multisystem CMCs, particularly those affecting the respiratory, cardiovascular, or gastrointestinal systems, had second-season RSV hospitalization rates that were at least 2-fold higher than the rate among all children in the first season. Second-season rates among children with Down syndrome or those who were born prematurely (<28 weeks of gestation) were 5-fold higher than for all children in the first season.

**Conclusions and Relevance:**

This population-based retrospective cohort study identified specific groups of higher-risk children with CMCs who could most benefit from prophylaxis with long-acting monoclonal antibodies in their first and second RSV seasons. This study supports expanded eligibility criteria for long-acting monoclonal antibody prophylaxis.

## Key Points

**Question:**

Which children with chronic medical conditions (CMCs) are at higher risk for respiratory syncytial virus (RSV) hospitalization during their first 2 RSV seasons?

**Findings:**

In this cohort study, children with CMCs exhibited high RSV hospitalization rates throughout the first 2 seasons. Children with CMCs affecting multiple body systems, such as respiratory, cardiovascular, and gastrointestinal conditions; extreme prematurity; or Down syndrome, had the highest rates of RSV hospitalization.

**Meaning:**

This study identified specific groups of children with CMCs who may benefit from prophylaxis with long-acting monoclonal antibodies in their first and second RSV season.

## Teaser

This cohort study investigates the association of pediatric chronic medical conditions with risk of hospitalization for respiratory syncytial virus (RSV) during children's first 2 RSV seasons.

## Introduction

Respiratory syncytial virus (RSV) is the leading cause of lower respiratory tract infections (LRTIs) among young children.^1^ For the past 3 decades, the only available preventive intervention was palivizumab, a short-lived monoclonal antibody administered monthly during the RSV season to a restricted group of children at high risk.^2,3^ In 2023, two new products were approved for RSV prevention in young children: RSVpreF, a vaccine administered during pregnancy, and nirsevimab, a single-dose long-acting monoclonal antibody administered to the child.^4,5^ Recognizing the high disease burden caused by RSV, National Immunization Technical Advisory Groups (NITAGs) in North America and Europe recommended universal RSV immunization for infants entering their first RSV season instead of the restricted use of palivizumab only for children at high risk.^6,7,8,9^ They have also recommended nirsevimab for children who remain at increased risk during a second RSV season and those who are at high risk in their first season, regardless of maternal RSV vaccination. Other NITAGs recommended nirsevimab only for children at high risk for up to 2 RSV seasons, including those who traditionally received palivizumab.^10^ However, all these recommendations remain imprecise due to a lack of data on high-risk groups outside of children eligible for palivizumab or beyond a child’s first RSV season.

Approximately 1% to 2% of children require hospitalization due to RSV in the first year of life. The highest rates occur within 3 months after birth for healthy, full-term infants.^1,11^ Medical fragility and reduced exposure to RSV after prolonged hospitalizations early in life may defer the risk from RSV infection beyond a first season among children with chronic medical conditions (CMCs).^12,13,14^ This persistent risk has been studied in specific groups of children, including those born preterm or born with chronic lung disease or hemodynamically significant congenital heart disease.^15,16^ However, we lack data for many other groups of children with CMCs.

In most countries, RSV antibody prophylaxis is administered on a seasonal basis. However, to our knowledge, no studies have broadly examined RSV disease rates in children with CMCs at the population level by season of exposure rather than age. This knowledge gap complicates the utility of data for refining public health recommendations in clinical settings. This highlights the need for studies that pragmatically quantify RSV outcomes among children with CMCs based on their eligibility at the start of the RSV season. This study aimed to identify groups of children with CMCs who were at higher risk of RSV hospitalization during their first and second RSV seasons.

## Methods

### Study Design, Setting, and Participants

This retrospective population-based cohort study was conducted among children born in British Columbia, Canada, between April 1, 2013, and March 31, 2023, with or without a CMC (main exposure). British Columbia is the westernmost province of Canada, with a population of 5.7 million in 2024 and approximately 42 000 births per year on average over the past 5 years. Children were identified through the provincial health registry that includes all children enrolled in provincial public health care (approximately 99.9% of all live births in British Columbia). Children whose registration dates in the provincial health service plan could not be determined were excluded. Follow-up began at the start of a child’s first RSV season (defined below) if born before the season or at birth if born during the season until the day preceding their third RSV season or April 1, 2024 (last day of available data), whichever came first (eFigure 1 in Supplement 1). Respiratory syncytial virus seasons were defined between October 1 and March 31, based on epidemic trends observed in British Columbia over the prior 15 years,^17^ with data collected through September 30 to capture all events in periods of low RSV activity. This study was approved by the University of British Columbia Children’s & Women’s Research Ethics Board, which also waived the requirement for participants’ consent, as no identifiable data were required or used. This study follows the Strengthening the Reporting of Observational Studies in Epidemiology (STROBE) reporting guideline for cohort studies.

### Measures

The exposure variable was the presence of CMCs, based on a modified Pediatric Complex Chronic Condition classification, which uses *International Statistical Classification of Diseases and Related Health Problems, Tenth Revision* (*ICD-10*), codes for conditions that “can be reasonably expected to last at least 12 months… severe enough to require specialty pediatric care and probably some period of hospitalization in a tertiary care center.”^18^ All CMCs diagnosed within the first 2 years of life were included. Once a CMC was diagnosed, the child was classified in the CMC group for the entire follow-up period. The CMCs in modified Pediatric Complex Chronic Condition classification (ie, diagnoses for *ICD-10* code list and CMC classification; see eTables 1 and 2 in Supplement 2 for more details) were grouped in 10 categories also by body system, congenital anomalies, or genetic anomalies (eTable 1 in Supplement 1).

The primary outcome was RSV LRTI–related hospitalization by *ICD-10* codes (eMethods in Supplement 1), documented either as the most responsible or contributory diagnosis. Using other Canadian data, this outcome had a sensitivity and specificity exceeding 95%.^19^ An internal validation was also conducted, showing no differential misclassification between the CMC and no CMC groups (eFigure 2 in Supplement 1), and a specificity of 97.9% (95% CI, 97.2%-98.1%) and a sensitivity of 80.7% (95% CI, 79.4%-82.0%) compared with positive RSV test results (eMethods in Supplement 1). Readmissions within 30 days of the initial RSV-LRTI event were considered part of the same episode.

Secondary outcomes included pediatric intensive care unit (PICU) admission, lengths of hospital and PICU admissions (in days), use of invasive mechanical ventilation (using *ICD-10* Canadian Institute of Health Information intervention codes: 1.GZ.31.CA-ND and 1.GZ.31.CR-ND), and in-hospital mortality.

Other variables included same-season palivizumab administration, sex, month of birth, prematurity at birth, neonatal intensive care unit stay (in days), and urban-rural classification of place of residence (based on British Columbia’s urban-rural classification). The study preceded the availability of nirsevimab or adult RSV vaccines in Canada.

### Data Sources

Data were obtained through the Provincial Health Service Authority’s Platform for Analytics and Data (PANDA).^20,21^ The integrated PANDA platform provides data, governance, tools, and services to support reporting and analytics of health data in British Columbia for users and researchers across the Provincial Health Service Authority. Details on sources and data linkage are detailed in eTable 2 in Supplement 1.

### Statistical Analysis

All primary analyses were prespecified. As this is a population-based study, no sample size calculations were performed. Baseline cohort characteristics and outcomes were summarized by the exposure variable (children with or without CMCs) using counts and percentages for categorical variables and median (IQR) values for continuous variables. Primary and secondary outcomes were summarized as proportions of RSV-LRTI hospitalizations, PICU admission and mechanical ventilation for RSV-LRTI, and median length of hospitalization or PICU admission. Outcomes occurring in the second RSV season were compared between children with and those without CMCs using the Mann-Whitney-Wilcoxon test for continuous variables and the χ^2^ test for proportions. Incidence rates (IRs) for RSV hospitalizations were estimated as number of events per person-years (to account for potential loss to follow-up) with 95% CIs. Children who died or moved out of British Columbia before the end of follow-up were censored at the date of loss to follow-up. Cell sizes with fewer than 6 events were reported as “<6” as mandated by the data stewardship and British Columbia Ministry of Health data reporting practices.

Incidence rates were first estimated by monthly epochs as a function of postnatal age (0-24 months of age), comparing children with and without CMCs. Season-specific IRs were then estimated comparing second-season children with CMCs with all first-season children (with or without CMCs), as the main population recommended to be covered by universal RSV immunization programs. Adjusted season-stratified IRs, IR ratios, and rate differences between groups were estimated using a Poisson generalized estimating equation model with an exchangeable correlation structure. Generalized estimating equations accounted for repeated (over 2 seasons) observations from the same children and included an offset term for follow-up time, sex, and palivizumab administration in each season. Sex assigned at birth was treated as a confounder due to its established associations with both CMCs and risk of RSV-LRTI.^22^ Another model was generated adjusting for prematurity, categorized as birth at less than 28 completed weeks (less than 196 completed days) of gestation, 28 completed weeks’ or more but less than 37 completed weeks’ and 37 weeks’ gestational age or more. Prior RSV infection may decrease the risk of reinfection due to immunity acquisition or increase it through a confounder effect (eg, selecting more fragile, medically complex children). However, IRs were not adjusted for this variable as RSV infections not requiring hospitalization would be undiagnosed (not routinely tested). Moreover, multiple RSV-LRTI hospitalizations were expected to be negligible based on previous studies.^23^ In subgroup analyses, season-stratified IRs were further stratified by CMC body system and congenital or genetic anomalies (detailed in eTable 1 in Supplement 1), by number of body systems involved and selected conditions, all compared with all childrens’ first-season rates. Because prematurity less than 28 weeks of gestation frequently occurs concurrently with other CMCs,^24^ a subgroup analysis was also conducted excluding those children.

To address potential measurement bias from undercoding of RSV cases, a sensitivity analysis was conducted using an expanded outcome definition: primary outcome definition plus all respiratory-related hospital admissions (where the most responsible *ICD-10* diagnosis code begins with “J”) along with a positive RSV test result within 7 days of admission. Atypical RSV epidemic trends were reported in British Columbia due to the COVID-19 pandemic, from March 2020 to 2023.^17,25,26^ To account for these changes in RSV epidemiology, a sensitivity analysis was conducted excluding children born after April 2018. All analyses were conducted using R statistical software, version 4.3.1 (R Project for Statistical Computing). All *P* values were from 2-sided tests, and results were deemed statistically significant at *P* < .05.

## Results

### Cohort Characteristics

In total, 431 996 children were assessed for eligibility. The final cohort consisted of 431 937 children (32 959 [7.6%] born at <37 weeks’ gestation; 222 207 boys [51.4%] and 209 726 girls [48.6%]) after the exclusion of 59 children, whose timing of registration to the British Columbia health insurance plan could not be determined. The median follow-up time was 728 days (IQR, 642-729 days), with 1098 of 431 937 (0.3%) of the children lost to follow-up before the start of their second season (median lost to follow-up time, 4 days [IQR, 0-92 days] after birth). Therefore, 431 937 children were included in the first-season analyses and 430 839 children (99.7%) were included in the second-season analyses (eFigure 3 in Supplement 1). Baseline characteristics for children with and those without CMCs are shown in Table 1.^18,27^ Overall, 5.9% (25 452 of 431 937) of children received diagnoses of at least 1 of 1116 distinct CMCs within their first 2 years of life (94.2% [23 984 of 25 452] received a diagnosis within 30 days of life). Sex distribution, residential remoteness from urban centers, and median follow-up time were similar between the 2 groups. A higher proportion of children with CMCs than those without CMCs were born before 37 weeks of gestation (27.4% [6982 of 25 452] vs 6.4% [25 977 of 406 485]). Children with CMCs had greater attrition than those without CMCs (2.9% [730 of 25 452] vs 0.1% [368 of 406 485]; mainly due to deaths) and a greater proportion of neonatal intensive care unit admissions compared with healthy children.

**Table 1.** Baseline Characteristics Comparing Children With vs Without CMCs^a^

Characteristic	Children, No. (%)		
	Overall (N = 431 937)	CMCs (n = 25 452)	No CMCs (n = 406 485)
Sex at birth			
Female	209 726 (48.6)	12 598 (49.5)	197 128 (48.5)
Male	222 207 (51.4)	12 853 (50.5)	209 354 (51.5)
Gestational age at birth			
≥37 wk	398 978 (92.4)	18 470 (72.6)	380 508 (93.6)
28-36 wk + 6 d	31 589 (7.3)	5612 (22.0)	25 977 (6.4)
<28 wk	1370 (0.3)	1370 (5.4)	0
Birth admission to NICU (>72 h)	13 894 (3.2)	5103 (20.0)	8791 (2.2)
Length of birth admission to NICU, median (IQR), d	9 (5-20)	14 (7-31)	7 (5-14)
Place of residence at birth^b^			
Metropolitan	219 815 (51.0)	13 637 (53.7)	206 178 (50.8)
Urban	154 640 (35.9)	8918 (35.1)	145 722 (35.9)
Rural	53 424 (12.4)	2675 (10.5)	50 749 (12.5)
Remote	3253 (0.8)	176 (0.7)	3077 (0.8)
Received palivizumab in first RSV season	3027 (0.7)	2011 (7.9)	1016 (0.2)
Received palivizumab in second RSV season	293 (0.1)	278 (1.1)	15 (<0.01)

Abbreviations: CMCs, chronic medical conditions, based on the Pediatric Complex Chronic Condition classification^18^; NICU, neonatal intensive care unit; RSV, respiratory syncytial virus.

^a^ Missing data: sex was indeterminate for fewer than 6 individuals. For place of residence, data were missing for 805 individuals (46 children with CMCs and 759 children without CMCs). Percentages were based on number of children with available data.

^b^ Defined using the Statistics Canada index of remoteness.^27^

### RSV Hospitalization by Postnatal Age

**Figure 1.**
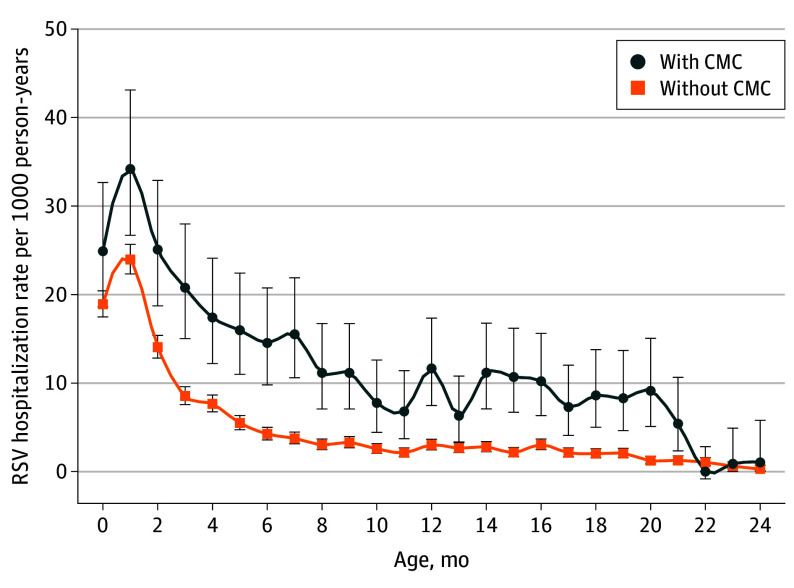
Respiratory Syncytial Virus (RSV) Hospitalization Rates by Postnatal Age Among Children With vs Without Chronic Medical Conditions (CMCs) Respiratory syncytial virus hospitalization incidence rates per 1000 person-years by monthly postnatal age period among children with CMCs. Error bars indicate 95% CIs.

In total, 4567 children (1.1%) experienced a combined total of 4592 hospitalizations (4320 with RSV-LRTI as the most responsible diagnosis). Respiratory syncytial virus hospitalizations rates among children with CMCs were higher compared with children without CMCs until 20 months of age (Figure 1), with sustained higher risk in the second year of life, particularly among those with CMCs involving the respiratory and gastrointestinal systems, and among children with Down syndrome or born preterm at less than 28 weeks of gestation (eFigure 4 in Supplement 1).

### Season-Stratified RSV Hospitalization Outcomes

In the season-stratified analyses, 0.8% of all children (3427 of 431 937) were hospitalized in their first RSV season and 0.8% of children with CMCs (204 of 24 723) were hospitalized in their second RSV season (Table 2).^18^ In the first season, the RSV hospitalization rate per 1000 person-years for children with CMCs was 15.9 (95% CI, 14.2-17.6) and for children without CMCs was 8.0 (95% CI, 7.7-8.3). In the second season, the RSV hospitalization rate per 1000 person-years for children with CMCs was 7.8 (95% CI, 6.7-8.8) and for children without CMCs was 2.2 (95% CI, 2.1-2.3) (see Table 3 for second-season data and eTable 3 in Supplement 1 for first-season data). The adjusted IRs for RSV hospitalizations were comparable between children with CMCs in their second season and all children in their first season, with no significant absolute risk difference (Table 3).^18^ The prespecified sensitivity analyses, either using expanded RSV outcome definitions including all respiratory *ICD-10* codes and a positive RSV test result within 7 days of admission, or restricted to prepandemic seasons (ie, children born before April 2018) yielded similar results (eTable 4 in Supplement 1). However, IRs were lower among children with CMCs in the second RSV season compared with children in the first RSV season when adjusting for prematurity (Table 3).^18^ Children with CMCs required longer hospital and PICU stays and more mechanical ventilation in their first 2 seasons compared with healthy children (Table 2^18^; eTable 3 in Supplement 1).

**Table 2.** Primary (RSV Hospitalizations) and Secondary Outcomes Between Children With vs Without CMCs by Season

Outcome	First RSV season, all children (N = 431 937)	Second RSV season	
		CMCs (n = 24 723)	No CMCs (n = 406 116)
Hospitalized for RSV-LRTI during that season, No. (% children)	3427 (0.8)	204 (0.8)^a^	936 (0.2)
1 RSV-LRTI episode, No. (% hospitalized)^b^	3411 (99.5)	202 (99.0)	929 (99.3)
2 RSV-LRTI episodes, No. (% hospitalized)^b^	16 (0.5)	<6	7 (0.7)
Age at first RSV-LRTI episode, median (IQR), mo	2.4 (1.1-4.7)	15.6 (12.6-18.3)	15.4 (12.6-18.4)
RSV hospitalization in previous season, No. (% hospitalized)	NA	13 (6.4)	22 (2.4)
Length of hospital stay, median (IQR), d	3 (2-5)	4 (2-6)^a^	2 (2-4)
PICU admission, No. (% hospitalized)^c^	413 (12.0)	29 (14.2)^a^	56 (5.9)
Length of PICU admission, median (IQR), d	4 (2-6)	3 (1-8)^a^	2 (1-4)
Mechanical ventilation, No. (% hospitalized)^d^	135 (3.9)	10 (4.9)^a^	11 (1.2)

Abbreviations: CMCs, chronic medical conditions, based on the Pediatric Complex Chronic Conditions classification^18^; PICU, pediatric intensive care unit; RSV, respiratory syncytial virus; RSV-LRTI, RSV-related lower respiratory tract infection based on *International Statistical Classification of Diseases and Related Health Problems, Tenth Revision*, codes.

^a^ *P* < .001 comparing the CMC vs no CMC groups in the second season, using the Mann-Whitney-Wilcoxon test for lengths of hospital stay and PICU stay and with the χ^2^ test for proportions of PICU admission and need for mechanical ventilation.

^b^ Episode refers to an initial RSV-LRTI hospitalization and any subsequent readmissions due to RSV-LRTI within 30 days of initial admission.

^c^ Pediatric intensive care unit admission defined as admissions involving the intensive care unit at British Columbia Children’s Hospital and Victoria General Hospital.

^d^ Mechanical ventilation based on the Canadian Institute of Health Information intervention codes 1.GZ.31.CA-ND and 1.GZ.31.CR-ND.

**Table 3.** Risk of RSV-LRTI Hospitalization Among Children With CMCs in the Second Season vs First Season

Variable	Incidence rate per 1000 person-years (95% CI)^a^		
	First RSV season, all children (N = 431 937)	Second RSV season	
		Children with CMCs (n = 24 723)	Children without CMCs (n = 406 116)
RSV hospitalizations, No.^b^	3443	206	943
Unadjusted hospitalization	9.1 (8.8 to 9.4)	8.4 (7.3 to 9.6)	2.3 (2.2 to 2.5)
Adjusted for sex and same-season palivizumab administration			
RSV hospitalization	8.5 (8.2 to 8.8)	7.8 (6.7 to 8.8)	2.2 (2.1 to 2.3)
Absolute risk difference (95% CI) per 1000 person-years	0 [Reference]	−0.7 (−1.8 to 0.4)	−6.3 (−6.6 to −6.0)
Incidence rate ratio (95% CI)	1 [Reference]	0.9 (0.8 to 1.1)	0.3 (0.2 to 0.3)
Adjusted for sex, same-season palivizumab, and prematurity			
RSV hospitalization	8.5 (8.2 to 8.8)	6.2 (5.4 to 7.1)	2.2 (2.1 to 2.4**)**
Absolute risk difference (95% CI) per 1000 person-years	0 [Reference]	−2.3 (−3.9 to −1.4)	−6.3 (−6.6 to −6.0)
Incidence rate ratio (95% CI)	1 [Reference]	0.7 (0.6 to 0.8)	0.3 (0.2 to 0.3)

Abbreviations: CMCs, chronic medical conditions, based on the Pediatric Complex Chronic Conditions classification^18^; RSV, respiratory syncytial virus; RSV-LRTI, RSV-related lower respiratory tract infection based on *International Statistical Classification of Diseases and Related Health Problems, Tenth Revision*, codes.

^a^ Adjusted incidence rates were calculated using a modified Poisson regression model.

^b^ Including multiple RSV-LRTI hospitalizations per child, if the episode occurred more than 30 days apart.

### **R**SV Hospitalizations by Body Systems Chronically Affected

**Figure 2.**
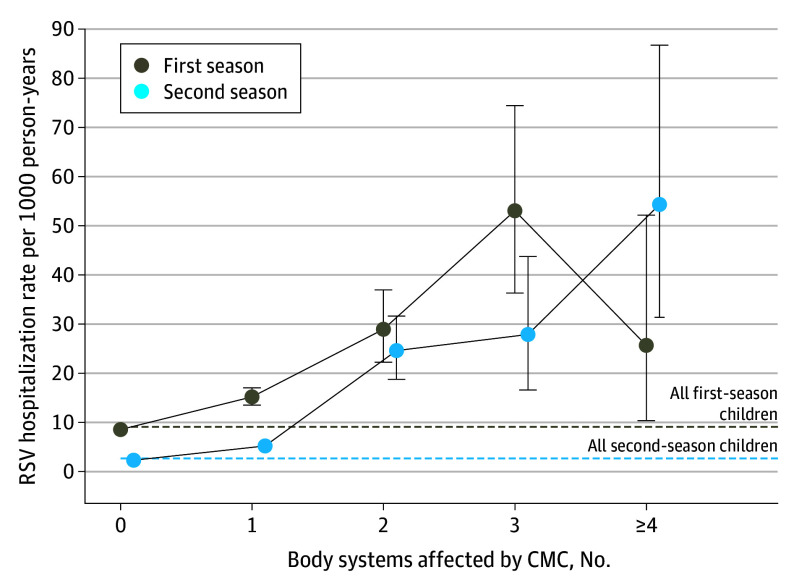
Respiratory Syncytial Virus (RSV) Hospitalization Rates Among Children With Chronic Medical Conditions (CMCs) by Number of Body Systems Affected Respiratory syncytial virus hospitalization incidence rates per 1000 person-years. Horizontal lines indicate the rate in all children in their first RSV season (brown) or second RSV season (blue). Number of body systems are based on number of different CMC categories affecting each individual, according to the modified Pediatric Complex Chronic Conditions classification using *International Statistical Classification of Diseases and Related Health Problems, Tenth Revision* (*ICD-10*), codes diagnosed within the first 2 years of age (corresponding to “Category” in eTable 1 in Supplement 1). Error bars indicate 95% CIs. Data points were slightly shifted in the graph to avoid overlapping.

A network analysis showed body systems that were concurrently involved among children with CMCs in this cohort (eFigure 5 in Supplement 1), namely, neonatal, respiratory, genetic, and neurologic conditions. In both seasons, the number of body systems affected showed a dose-response association with RSV hospitalization rates among children with CMCs (Figure 2). Consequently, rates among children with CMCs in season 2 exceeded rates among children overall in season 1 when at least 2 body systems were affected. Children with Down syndrome had significantly higher RSV hospitalization rates even in the absence of other specific CMCs (eFigure 6 in Supplement 1).

Second-season hospitalization rates among children with CMCs involving the respiratory, cardiovascular, and gastrointestinal systems or those with a congenital anomaly were 2-fold higher than rates among children overall in season 1 (eFigure 7 in Supplement 1). However, the difference in second-season RSV hospitalizations rates among children with CMCs involving a congenital anomaly was no longer significantly different compared with children in season 1 after exclusion of children born before 28 weeks’ gestation (eFigure 8 in Supplement 1).

### RSV Hospitalizations for Specific CMCs

Second-season RSV hospitalization rates among children with primary lung disease, cardiac conduction disorders or dysrhythmias, epilepsy syndromes, brain malformations, surgical gastrointestinal conditions, esophageal atresia with or without trachea-esophageal fistula, or genetic disorders (including Down syndrome); requiring a gastrostomy or assisted ventilation by tracheostomy; or with CMCs who were born preterm (<28 weeks’ gestation) exceeded overall first-season rates for all children (eFigure 9 in Supplement 1). For example, children with Down syndrome or who were born prematurely had a 5-fold increase in the rates of hospitalization in their second season compared with first-season children.

## Discussion

This study provides robust, population-based estimates of RSV hospitalization rates across a wide range of CMCs diagnosable in the first 2 years of life. Children with CMCs had higher RSV hospitalization rates than healthy children during their first 2 RSV seasons. Hospitalization rates in the second RSV season among children with CMCs were similar to rates among all children in their first season. Therefore, long-acting monoclonal antibody prophylaxis may be warranted for a second season among children with CMCs if a universal monoclonal antibody program is considered warranted during the first season. A caveat is that prior randomized clinical trials primarily included children in their first RSV season and a restricted number of children who remained at high risk during their second season.^28^ Although pharmacologic data supported effectiveness in the high-risk group in the second season, observational effectiveness studies are needed to confirm the anticipated benefit on clinical outcomes.^29^ Second, the data identified that children with CMCs remained at high risk of RSV hospitalization throughout the first 2 seasons, especially those with Down syndrome, multisystem CMCs, or conditions affecting the respiratory, cardiovascular, or gastrointestinal systems, particularly when associated with extreme prematurity (<28 weeks’ gestational age). Given that maternal antibodies wane and the lack of evidence that protection from maternal antibodies persists beyond 6 months, these children would likely benefit from a long-acting monoclonal RSV antibody after maternal RSV vaccination, particularly if they were born before the start of the RSV season.

With the increasing availability of population-level administrative datasets, evidence has grown to identify additional groups of children who face at least double the risk of RSV hospitalization compared with healthy children.^13,14,22,30^ Kristensen et al^13^ conducted a population-based analysis that confirmed higher RSV hospitalization rates among children with cystic fibrosis and Down syndrome. Moreover, they highlighted additional conditions—including gastrointestinal, neuromuscular, and genitourinary disorders—associated with a higher burden of disease among children aged 0 to 23 months. A nationwide study in Japan, which included children up to 5 years of age, demonstrated a direct association between the number of CMCs and the risk of severe RSV outcomes, suggesting an additive risk with the presence of multiple CMCs.^30^ The present study validates these previous findings using a season-based analysis, broadening condition-associated RSV disease rates beyond specific risk groups, such as children traditionally eligible for palivizumab.

Respiratory syncytial virus hospitalization rates in this British Columbia cohort were marginally lower than rates reported in some other countries or Canadian provinces.^1,19,31^ These lower rates could be due to differences in the follow-up period used to calculate estimates. Other possible factors include geographical and population factors contributing to varying burden of disease.^32^ Understanding the regional burden of RSV disease is critical to inform local RSV immunization guidelines. In regions with high IRs, a universal RSV prophylaxis program may be warranted. However, in jurisdictions with a lower burden of disease, targeted programs for children at high risk in their second RSV season may be more cost-effective. In addition, overlooking differences in RSV outcomes between CMC groups may underestimate the relative cost-effectiveness of high-risk vs universal immunization programs.^33,34,35^

### Limitations

This study has some limitations. First, the use of retrospective health administrative data may introduce biases resulting from variations in coding practices and potentially leading to misclassification of RSV cases. Missingness in the data may also lead to selection bias, underscoring the inherent limitations in conducting secondary analysis of health administrative data originally assembled for a different clinical purpose to draw conclusions for very specific disease groups or at the individual level. Second, the analysis did not account for the timing of CMC diagnoses (although most were identified before 30 days of age) or treatments received (eg, repair of a cardiac defect or need for tracheostomy). Likewise, while this study takes a pragmatic approach by categorizing CMCs, it does not account for condition severity—an analysis that would require a much larger sample size. Third, the frequent co-occurrence of extreme prematurity among children with CMCs makes it difficult to disentangle these 2 effects. Fourth, the database used for these analyses included only children registered for provincial health insurance at birth and thereby excluded those who were not registered for health insurance or who moved to the province later in their first year of life. Some of these children may belong to vulnerable populations at higher risk of severe RSV disease. Although they would represent a small number of children (<0.5% of births per year), caution is warranted when generalizing conclusions to populations where certain CMCs may be overrepresented. Fifth, while these data provide a strong foundation to inform policy, we acknowledge that formal recommendations on which children should receive RSV prophylaxis must also consider additional factors, including local implementation logistics, economic constraints, and the structure of existing RSV prevention programs.

## Conclusions

This cohort study provides comprehensive data on RSV hospitalization rates among groups of children with CMCs in their first and second RSV seasons. Although our findings highlight the heterogeneity of RSV risk, future studies will be needed to derive and validate clinical risk scores or to further stratify risk within broad diagnostic categories, such as differentiating hemodynamically significant cardiac lesions or severe bronchopulmonary dysplasia from less severe conditions. Our results aim to support these future efforts and inform data-driven, context-specific decision-making.

## Notes

Supplement 1. **eFigure 1.** Schematic of Cohort Design With Follow-Up Periods According to Month of Birth **eFigure 2.** *ICD-10* Groups for Respiratory-Related Admissions Within 2 Years of Life, Between Children With CMCs and Without CMCs (No CMC), Based on the Availability of RSV Testing Data **eFigure 3.** Flowchart of Cohort Participants **eFigure 4.** RSV Hospitalization Rates by Postnatal Age and Type of Chronic Medical Conditions **eFigure 5.** Network Plot Illustrating the Relationships Between Body Systems Affected in Children With Chronic Medical Conditions **eFigure 6.** RSV Hospitalization Incidence Rates by Number of Body Systems Affected **eFigure 7.** RSV Hospitalization Incidence Rates by Body Systems Affected in Children With Chronic Medical Conditions **eFigure 8.** RSV Hospitalization Rates by Body Systems in Children With Chronic Medical Conditions, Excluding Those Born <28 Weeks **eFigure 9.** RSV Hospitalization Incidence Rates by Specific Chronic Medical Conditions **eTable 1.** List of *ICD-10* Codes Included With Each CMC Subgroup **eTable 2.** Data Sources Used for Analysis **eTable 3.** RSV Hospitalizations Outcomes Between First-Season Children With or Without Chronic Medical Conditions (CMCs), by Season **eTable 4.** Sensitivity Analysis With Modified Main Outcome Definitions **eMethods.** Primary Outcome Validation (RSV Hospitalizations) **eTable 5.** Proportions of Hospitalized Children in Cohort, With Positive RSV Tests by Respiratory Diagnosis Categories Based on *ICD-10* Codes **eTable 6.** Sensitivity and Specificity (95% Confidence Interval in Brackets) With of Using RSV-LRI as a Most Responsible Primary Diagnosis Among Hospitalized Children, Compared to RSV Testing Results **eTable 7.** RSV Respiratory Testing Results in Children With a Most Responsible Diagnosis Other Than RSV-LRI, Stratified According to the Presence of RSV-LRI as a Secondary Admission Diagnosis **eFigure 10.** RSV Testing Results in Children Requiring Hospital Admissions With a Most Responsible Diagnosis in the Respiratory Category (JXX.X), by De-Identified Health Authorities in British Columbia (A, B, …) **eFigure 11.** RSV Testing Results in Children Requiring Hospital Admissions With a Most Responsible Diagnosis in the Respiratory Category (JXX.X), by Annual Season Period (September 1 to August 31 of the Following Year) **eFigure 12.** RSV Testing Results in Children Requiring Hospital Admissions With a Most Responsible Diagnosis in the Respiratory Category (JXX.X), by Age Groups **eFigure 13.** RSV Testing Results in Children With a Most Responsible Diagnosis Other Than RSV-LRI and RSV-LRI as a Secondary Admission Diagnosis

Supplement 2. **eTable 1.** ICD-10 Code List **eTable 2.** CMC Classification

Supplement 3.
Data Sharing Statement
