# Supplementary material for: RSV Hospital Admissions During the First 2 Seasons Among Children With Chronic Medical Conditions
Source: JAMA Netw Open. 2025 Jul 8;8(7):e2519410. doi: 10.1001/jamanetworkopen.2025.19410 (PMC12238895; doi:10.1001/jamanetworkopen.2025.19410)
Supplement: Supplement 1. — eFigure 1. Schematic of Cohort Design With Follow-Up Periods According to Month of Birth eFigure 2. ICD-10 Groups for Respiratory-Related Admissions Within 2 Years of Life, Between Children With CMCs and Without CMCs (No CMC), Based on the Availability of RSV Testing Data eFigure 3. Flowchart of Cohort Participants eFigure 4. RSV Hospitalization Rates by Postnatal Age and Type of Chronic Medical Conditions eFigure 5. Network Plot Illustrating the Relationships Between Body Systems Affected in Children With Chronic Medical Conditions eFigure 6. RSV Hospitalization Incidence Rates by Number of Body Systems Affected eFigure 7. RSV Hospitalization Incidence Rates by Body Systems Affected in Children With Chronic Medical Conditions eFigure 8. RSV Hospitalization Rates by Body Systems in Children With Chronic Medical Conditions, Excluding Those Born <28 Weeks eFigure 9. RSV Hospitalization Incidence Rates by Specific Chronic Medical Conditions eTable 1. List of ICD-10 Codes Included With Each CMC Subgroup eTable 2. Data Sources Used for Analysis eTable 3. RSV Hospitalizations Outcomes Between First-Season Children With or Without Chronic Medical Conditions (CMCs), by Season eTable 4. Sensitivity Analysis With Modified Main Outcome Definitions eMethods. Primary Outcome Validation (RSV Hospitalizations) eTable 5. Proportions of Hospitalized Children in Cohort, With Positive RSV Tests by Respiratory Diagnosis Categories Based on ICD-10 Codes eTable 6. Sensitivity and Specificity (95% Confidence Interval in Brackets) With of Using RSV-LRI as a Most Responsible Primary Diagnosis Among Hospitalized Children, Compared to RSV Testing Results eTable 7. RSV Respiratory Testing Results in Children With a Most Responsible Diagnosis Other Than RSV-LRI, Stratified According to the Presence of RSV-LRI as a Secondary Admission Diagnosis eFigure 10. RSV Testing Results in Children Requiring Hospital Admissions With a Most Responsible Diagnosis in the Respiratory Category (JXX.X), by De-Id [file jamanetwopen-e2519410-s001.pdf]

Supplementary Online Content

Viñeta Paramo M, Watts AW, Bone JN, et al. RSV hospital admissions during the first 2 seasons among children with chronic medical conditions. *JAMA Netw Open.* 2025;8(7):e2519410. doi:10.1001/jamanetworkopen.2025.19410

eFigure 1. Schematic of Cohort Design With Follow-Up Periods According to Month of Birth
eFigure 2. *ICD-10* Groups for Respiratory-Related Admissions Within 2 Years of Life, Between Children With CMCs and Without CMCs (No CMC), Based on the Availability of RSV Testing Data
eFigure 3. Flowchart of Cohort Participants
eFigure 4. RSV Hospitalization Rates by Postnatal Age and Type of Chronic Medical Conditions
eFigure 5. Network Plot Illustrating the Relationships Between Body Systems Affected in Children With Chronic Medical Conditions
eFigure 6. RSV Hospitalization Incidence Rates by Number of Body Systems Affected
eFigure 7. RSV Hospitalization Incidence Rates by Body Systems Affected in Children With Chronic Medical Conditions
eFigure 8. RSV Hospitalization Rates by Body Systems in Children With Chronic Medical Conditions, Excluding Those Born <28 Weeks
eFigure 9. RSV Hospitalization Incidence Rates by Specific Chronic Medical Conditions
eTable 1. List of *ICD-10* Codes Included With Each CMCs Subgroup
eTable 2. Data Sources Used for Analysis
eTable 3. RSV Hospitalizations Outcomes Between First-Season Children With or Without Chronic Medical Conditions (CMCs), by Season
eTable 4. Sensitivity Analysis With Modified Main Outcome Definitions
eMethods. Primary Outcome Validation (RSV Hospitalizations)
eTable 5. Proportions of Hospitalized Children in Cohort, With Positive RSV Tests by Respiratory Diagnosis Categories Based on *ICD-10* Codes
eTable 6. Sensitivity and Specificity (95% Confidence Interval in Brackets) With of Using RSV-LRI as a Most Responsible Primary Diagnosis Among Hospitalized Children, Compared to RSV Testing Results
eTable 7. RSV Respiratory Testing Results in Children With a Most Responsible Diagnosis Other Than RSV-LRI, Stratified According to the Presence of RSV-LRI as a Secondary Admission Diagnosis
eFigure 10. RSV Testing Results in Children Requiring Hospital Admissions With a Most Responsible Diagnosis in the Respiratory Category (JXX.X), by De-Identified Health Authorities in British Columbia (A, B, …)
eFigure 11. RSV Testing Results in Children Requiring Hospital Admissions With a Most Responsible Diagnosis in the Respiratory Category (JXX.X), by Annual Season Period (September 1 to August 31 of the Following Year)
eFigure 12. RSV Testing Results in Children Requiring Hospital Admissions With a Most Responsible Diagnosis in the Respiratory Category (JXX.X), by Age Groups
eFigure 13. RSV Testing Results in Children With a Most Responsible Diagnosis Other Than RSV-LRI and RSV-LRI as a Secondary Admission Diagnosis

This supplementary material has been provided by the authors to give readers additional information about their work.

© 2025 Viñeta Paramo M et al. *JAMA Network Open.*

eFigure 1. Schematic of Cohort Design With Follow-Up Periods According to Month of Birth

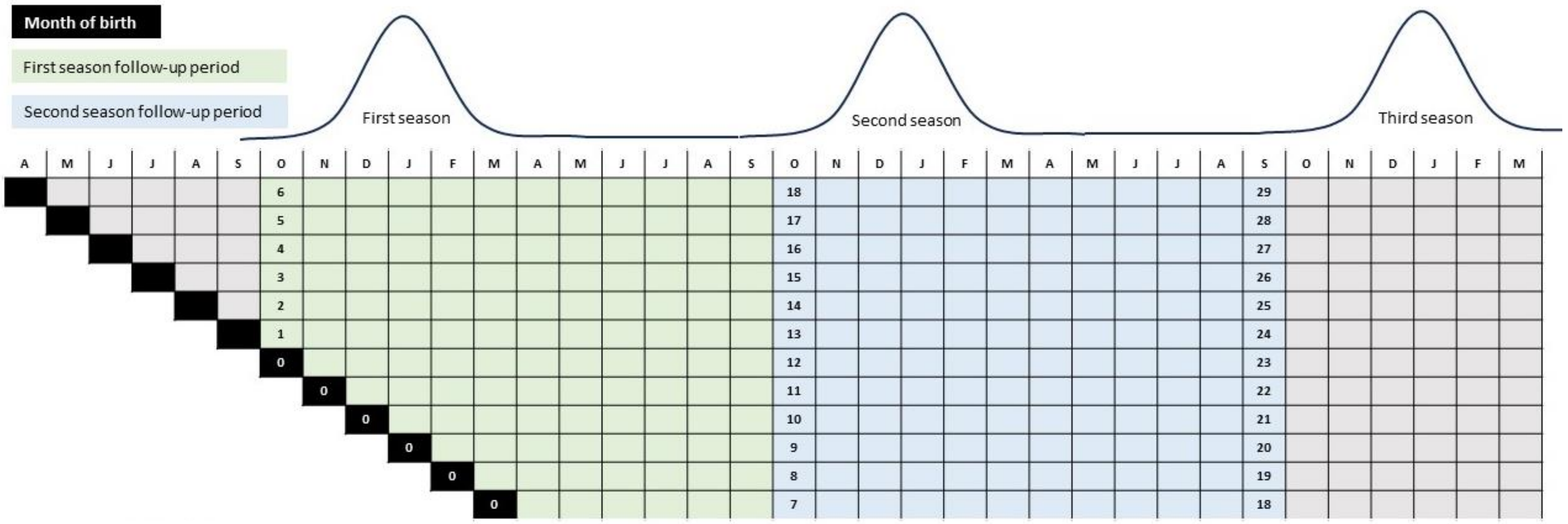

Season for a child started on October 1st (or at birth, if born between October 1st and March 31st), until September 30th of the following year. Numbers indicate the postnatal age in that specific month.

© 2025 Viñeta Paramo M et al. *JAMA Network Open.*

eFigure 2. *ICD-10* Groups for Respiratory-Related Admissions Within 2 Years of Life, Among Children With CMCs and Without CMCs (No CMC), Based on the Availability of RSV Testing Data

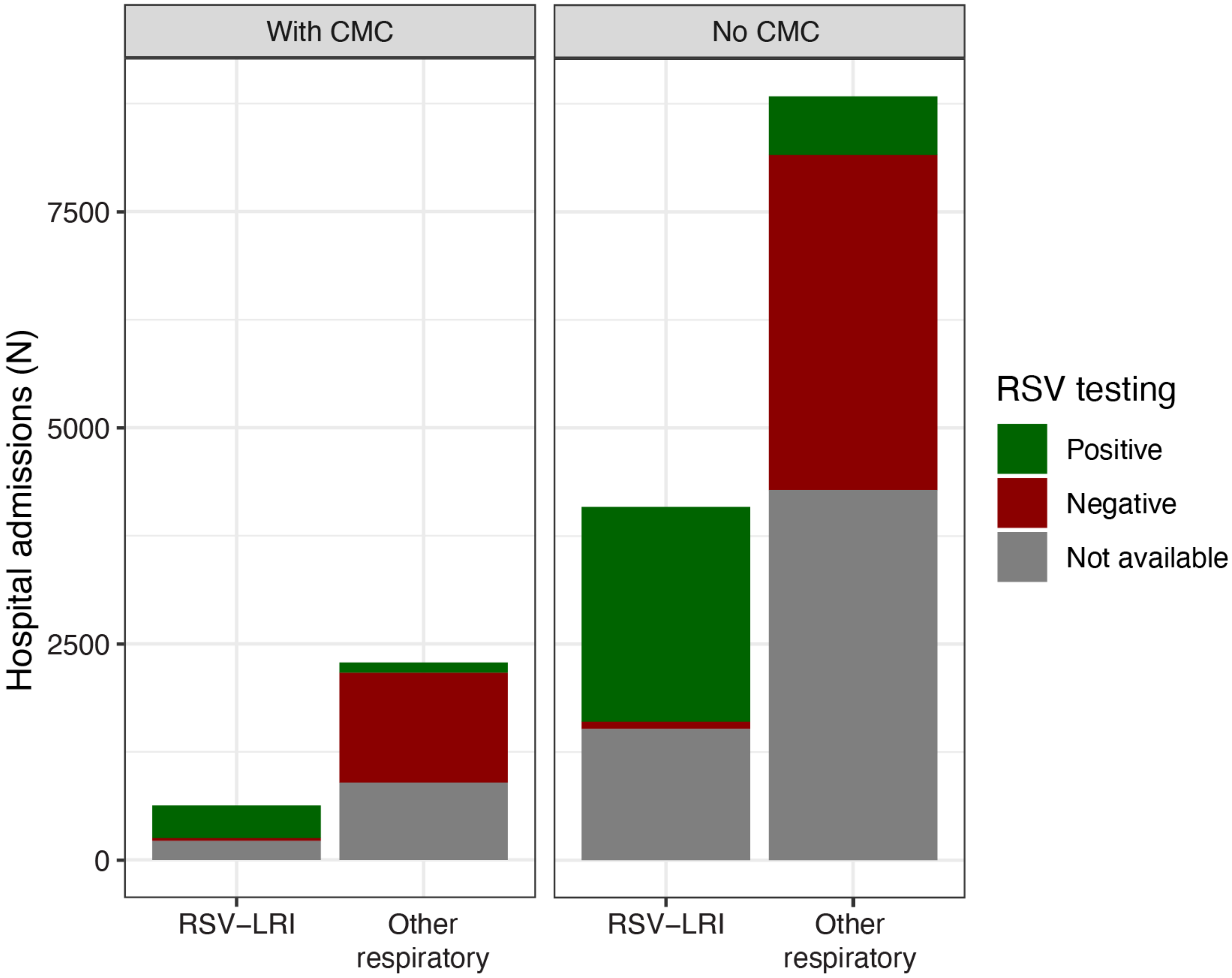

	CMCs		No CMC	
	RSV-LRI	Other	RSV-LRI	Other
Test available, n (% of N)	477 (58.7)	1,844 (52.5)	2,765 (61.0)	5,264 (43.9)
Test positive, n (% of N)	446 (54.9)	162 (4.6)	2,678 (59.1)	818 (6.8)

CMCs: Chronic medical conditions based on the Pediatric Complex Chronic Conditions classification[20]

“RSV-LRI” includes the following RSV-specific ICD-10 codes: J12.1 (*RSV pneumonia*), J20.5 (*Acute bronchitis due to RSV*), J21.0 (*Acute bronchiolitis due to RSV*), B97.4 (*Respiratory syncytial virus as the cause of diseases classified elsewhere*). “Other respiratory” includes any ICD-10 codes not included above that start with “J” (corresponding to diseases with respiratory symptoms).

© 2025 Viñeta Paramo M et al. *JAMA Network Open.*

eFigure 3. Flowchart of Cohort Participants

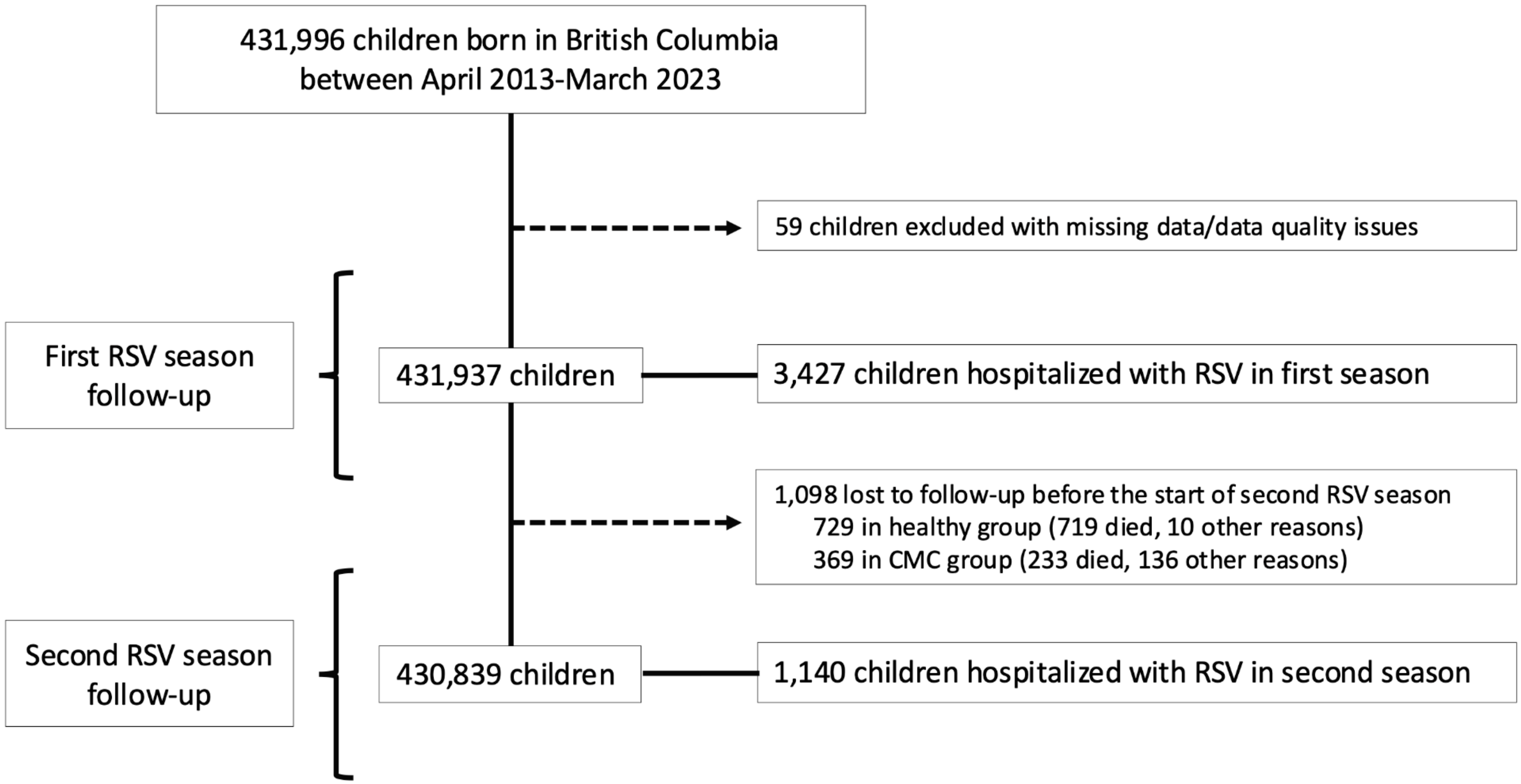

© 2025 Viñeta Paramo M et al. *JAMA Network Open.*

eFigure 4. RSV Hospitalization Rates by Postnatal Age and Type of Chronic Medical Conditions

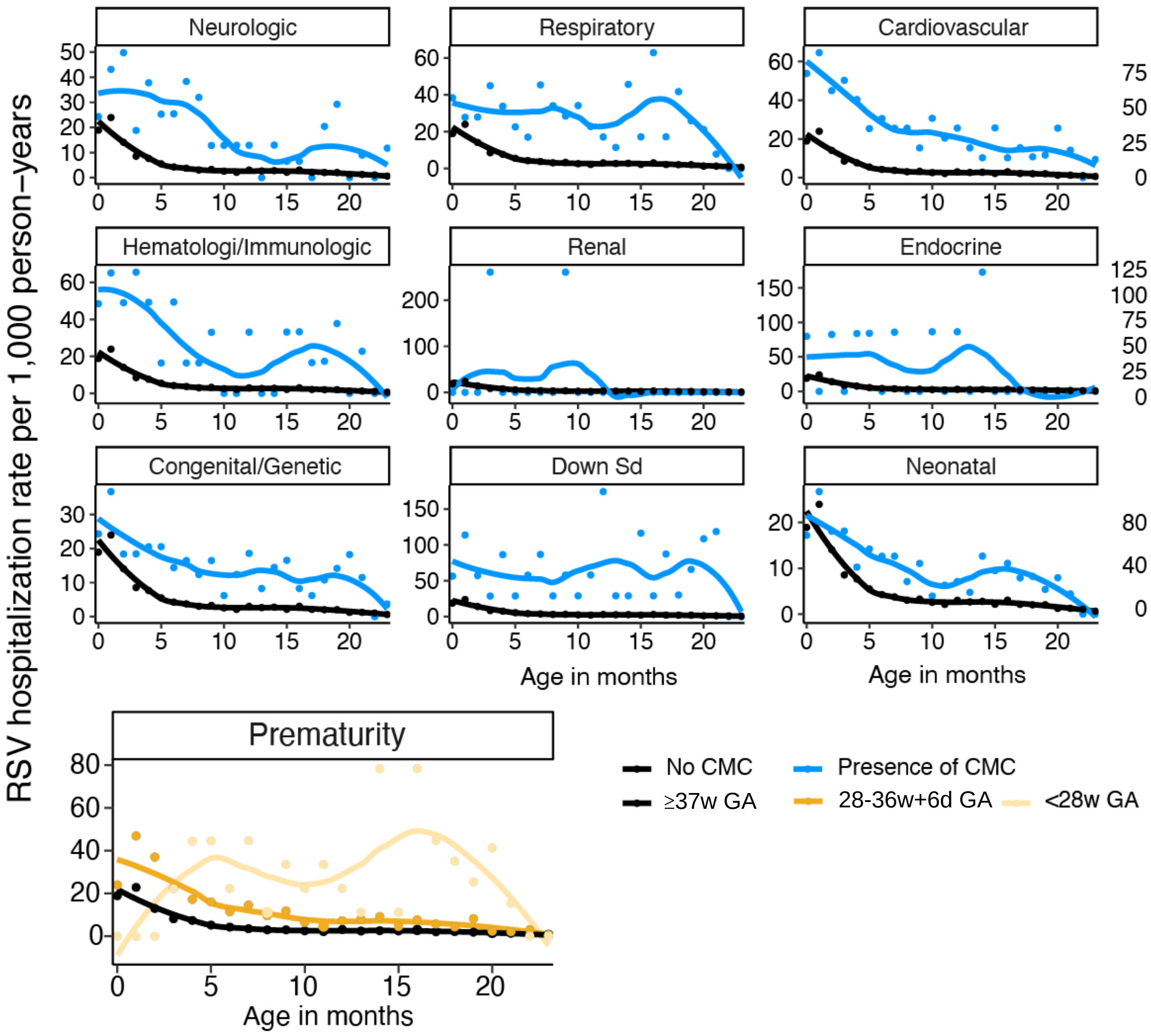

Points represent observed rates per 1000 person-years each month. Lines show smoothed estimates using locally weighted scatterplot smoothing (LOESS). Colors differentiate children with and without chronic medical conditions (CMCs), and panels correspond to CMC type or body system.

© 2025 Viñeta Paramo M et al. *JAMA Network Open.*

eFigure 5. Network Plot Illustrating the Relationships Between Body Systems Affected in Children With Chronic Medical Conditions

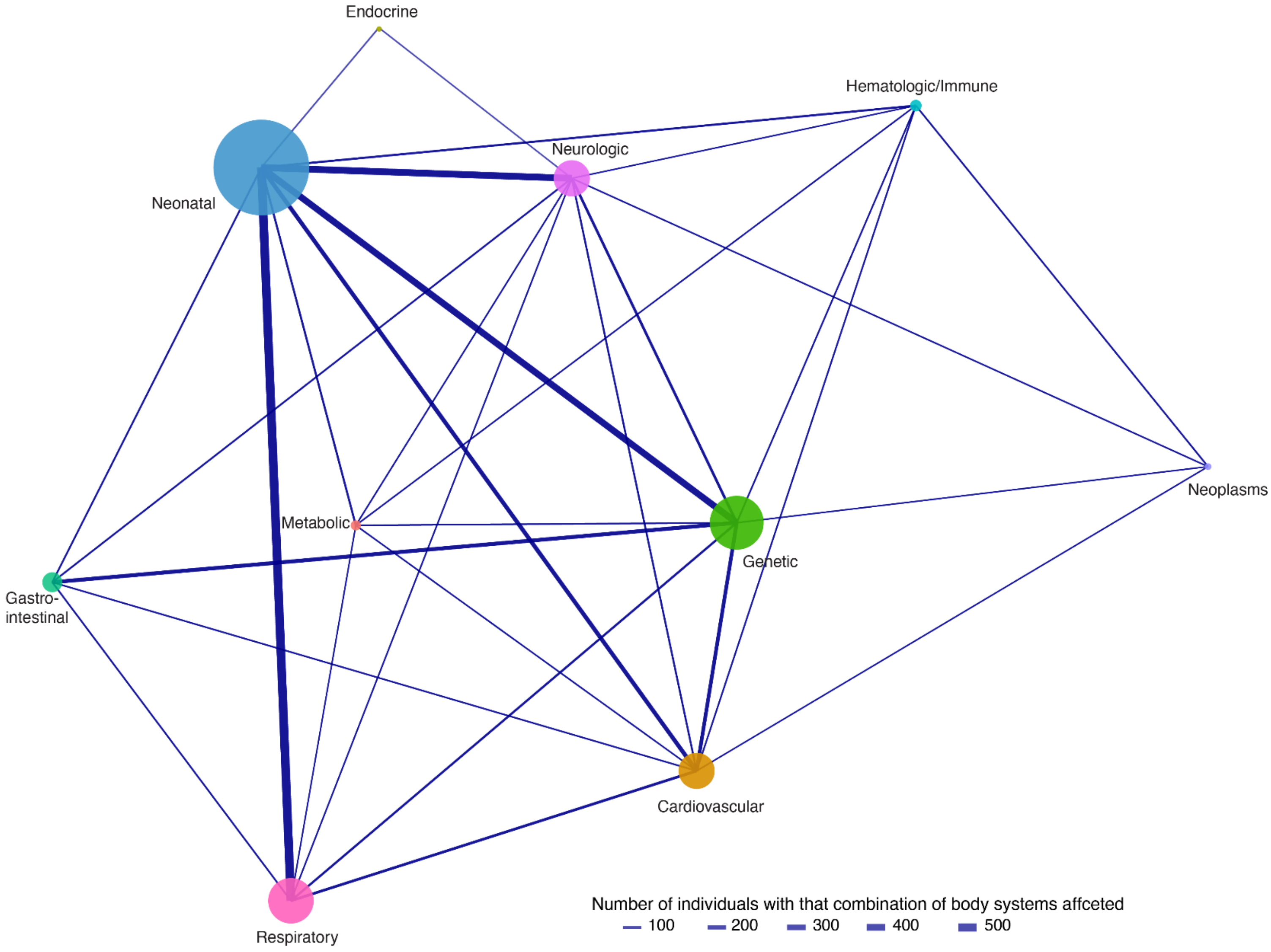

The co-occurrence of multiple CMCs affecting different body systems was visualized with a network of nodes representing each body system, proportional to the number of individuals experiencing a CMC in that system, and lines connecting body systems concurrently affected in same individuals. The thickness of the line corresponds to the number of individuals with concurrent involvement of the linked body systems.

© 2025 Viñeta Paramo M et al. *JAMA Network Open.*

eFigure 6. RSV Hospitalization Incidence Rates by Number of Body Systems Affected

Children with CMCs, and two or more body systems affected:

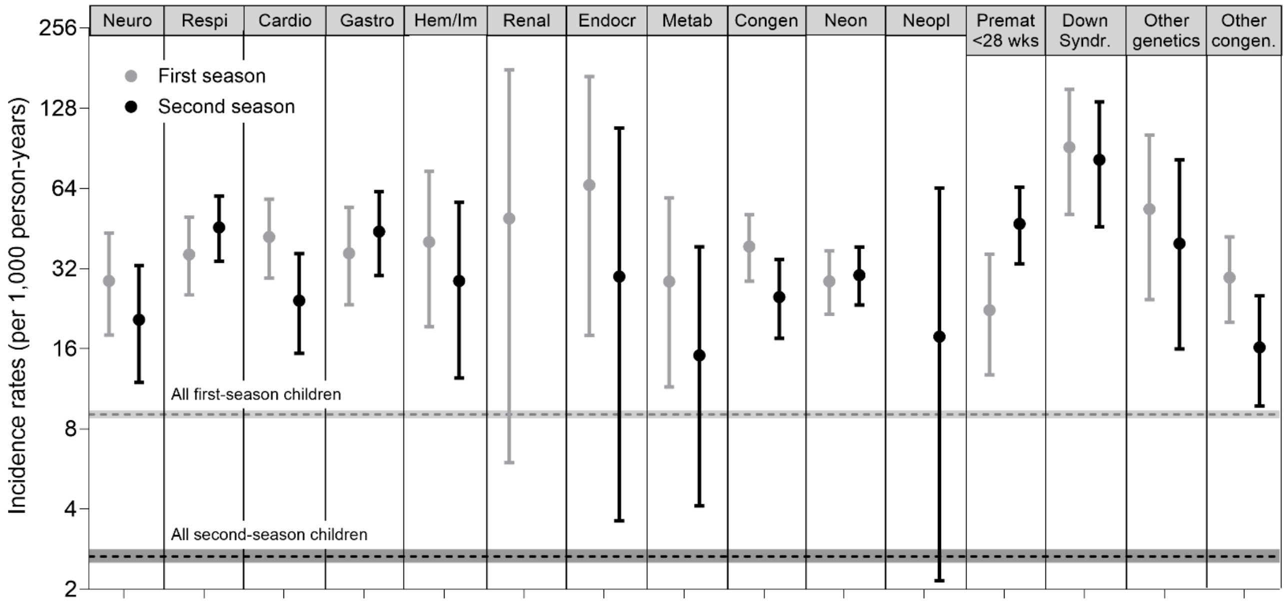

Children with CMCs, and only one body system affected:

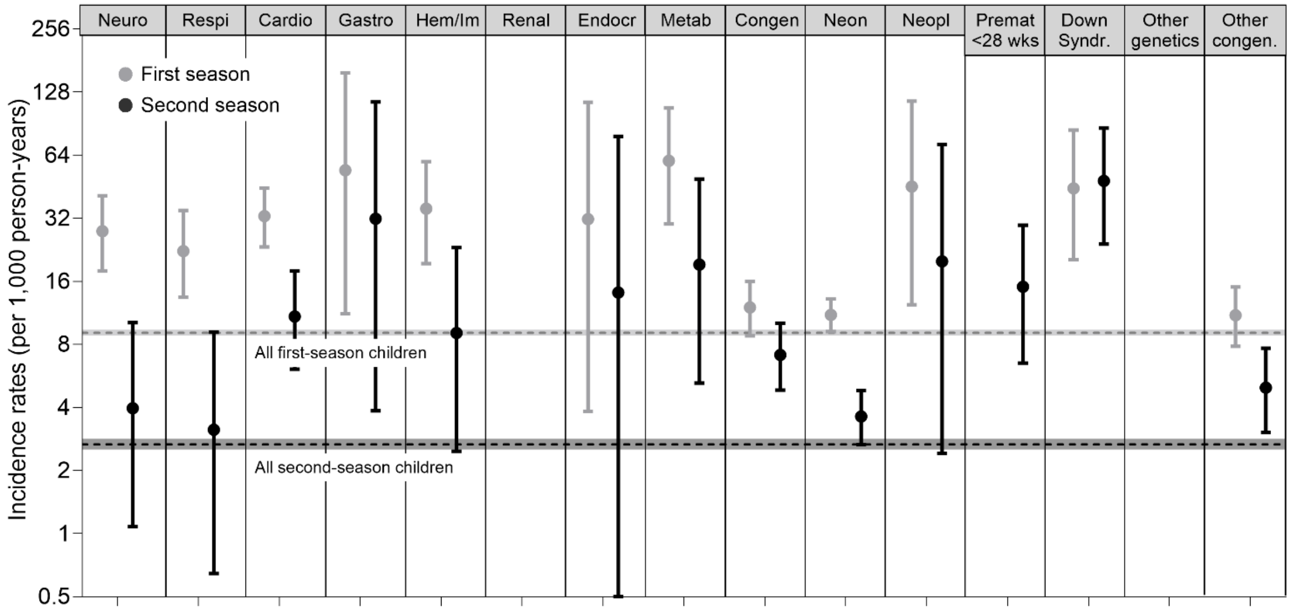

Incidence rates in children with two or more body systems affected (top graph) versus only one body system affected (top graph), with 95% confidence intervals. As prematurity <28 weeks was counted as a "body system" the categories in the bottom graph effectively exclude those children, except for the premature category itself which reciprocally shows IR for this condition in isolation. Horizontal lines indicate overall rates in all first (light grey dashed) or second (black dotted) season children. Some estimates are not shown as there were zero RSV hospitalization events. CMCs categories that are listed: neurologic, respiratory, cardiovascular, gastrointestinal, hematologic or immunologic, endocrine, metabolic, congenital or genetic, neonatal conditions and neoplasms.

© 2025 Viñeta Paramo M et al. *JAMA Network Open.*

eFigure 7. RSV Hospitalization Incidence Rates by Body Systems Affected in Children With Chronic Medical Conditions

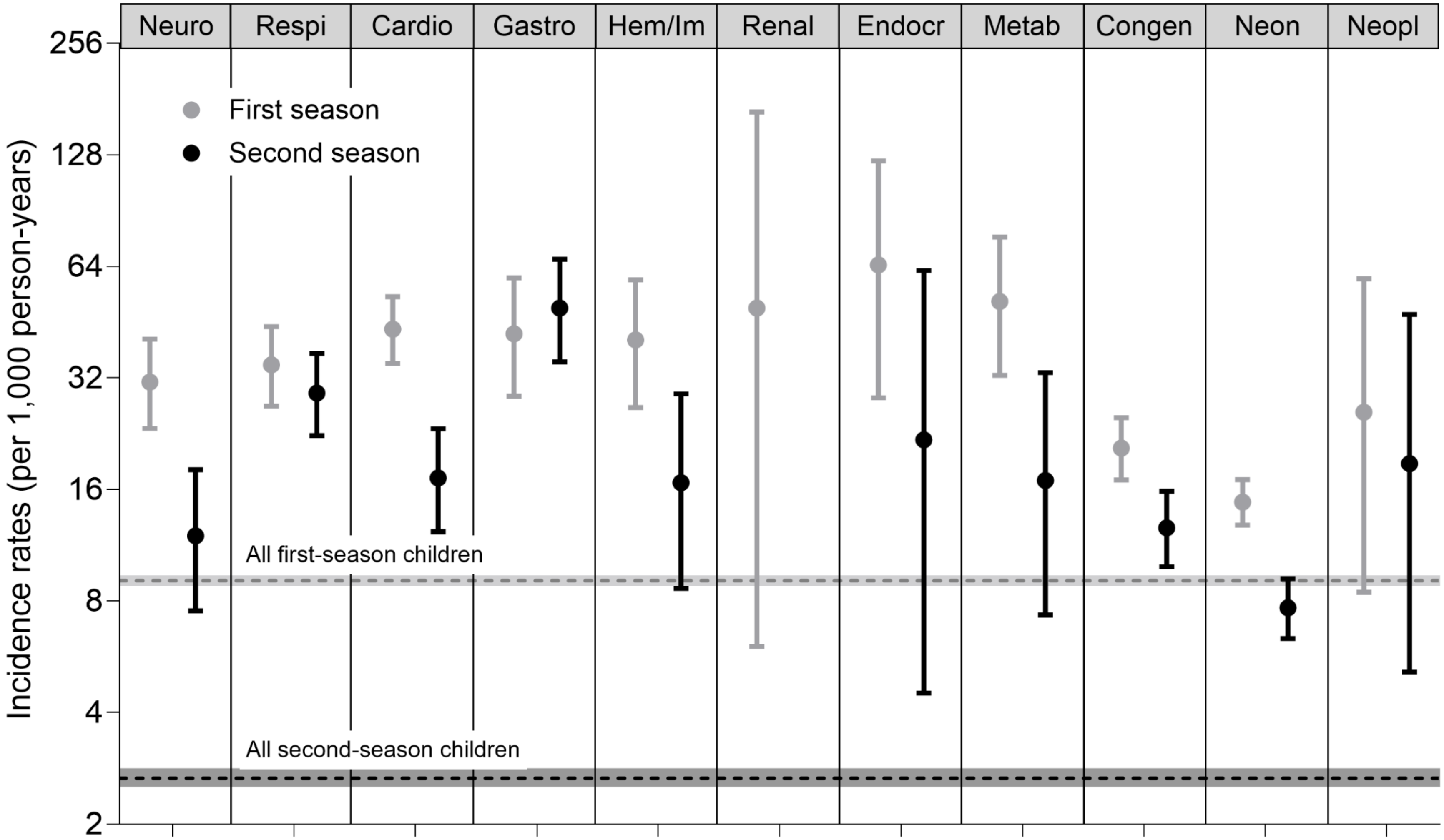

RSV hospitalization incidence rates per 1,000 person-years, with 95% confidence intervals. Horizontal lines indicate the rate in all first (light grey) or second (black) season children, with 95% confidence intervals shaded. Children were included if they had at least one ICD-10 code related to specific body system (Supplemental Table 1). Some estimates are not shown as there were zero RSV hospitalization events for this CMCs group and season. CMCs categories that are listed: neurologic, respiratory, cardiovascular, gastrointestinal, hematologic or immunologic, endocrine, metabolic, congenital or genetic, neonatal conditions and neoplasms.

© 2025 Viñeta Paramo M et al. *JAMA Network Open.*

eFigure 8. RSV Hospitalization Rates by Body Systems in Children With Chronic Medical Conditions, Excluding Those Born <28 Weeks

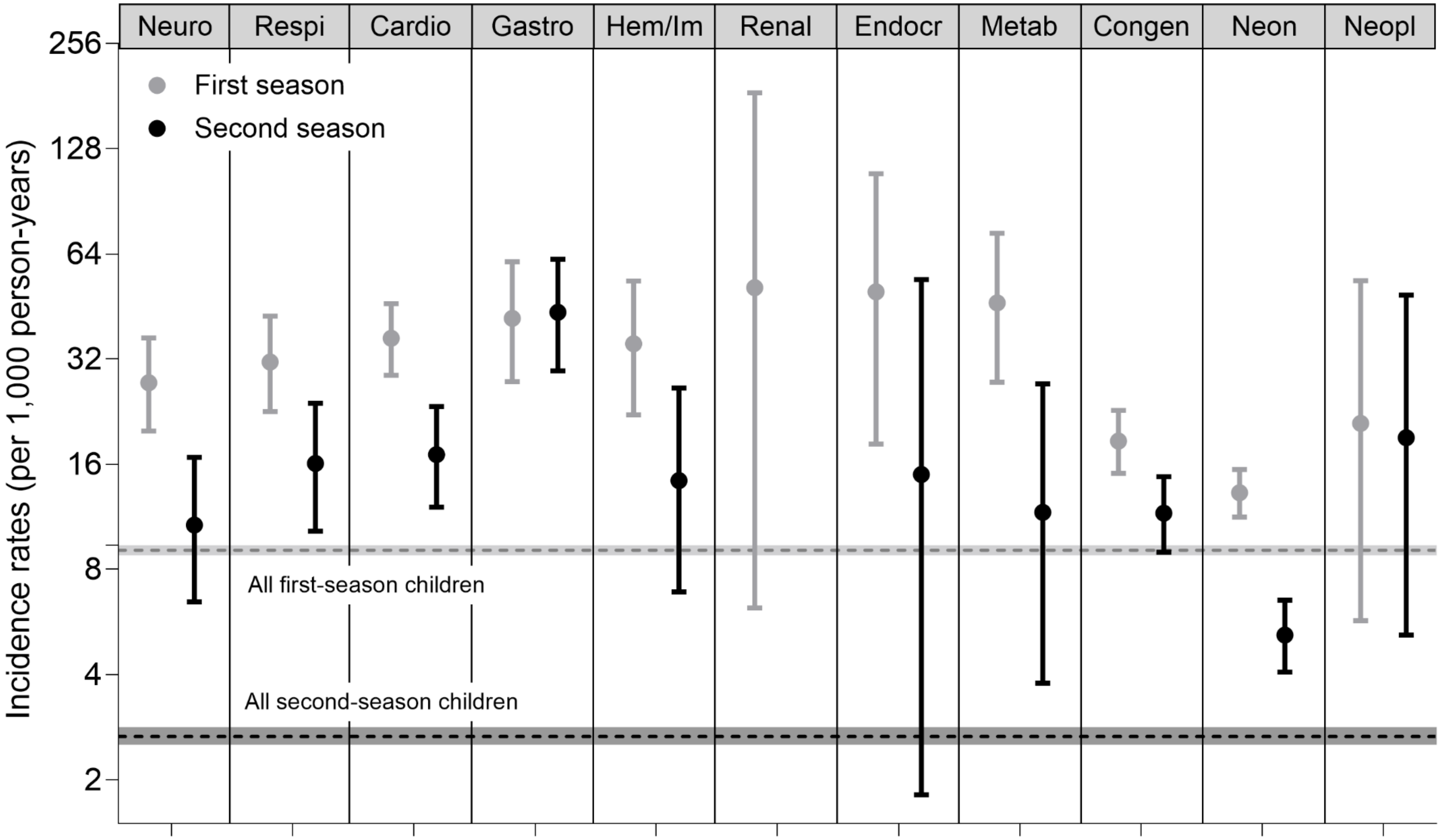

RSV hospitalization incidence rates per 1,000 person-years, with 95% confidence intervals. Horizontal lines indicate the rate in all first (light grey) or second (black) season children, with 95% confidence intervals shaded. Children were included if they had at least one ICD-10 code related to specific body system (Supplemental Table 1). Some estimates are not shown as there were zero RSV hospitalization events for this CMCs group and season. CMCs categories that are listed: neurologic, respiratory, cardiovascular, gastrointestinal, hematologic or immunologic, endocrine, metabolic, congenital or genetic, neonatal conditions and neoplasms.

© 2025 Viñeta Paramo M et al. *JAMA Network Open.*

eFigure 9. RSV Hospitalization Incidence Rates by Specific Chronic Medical Conditions

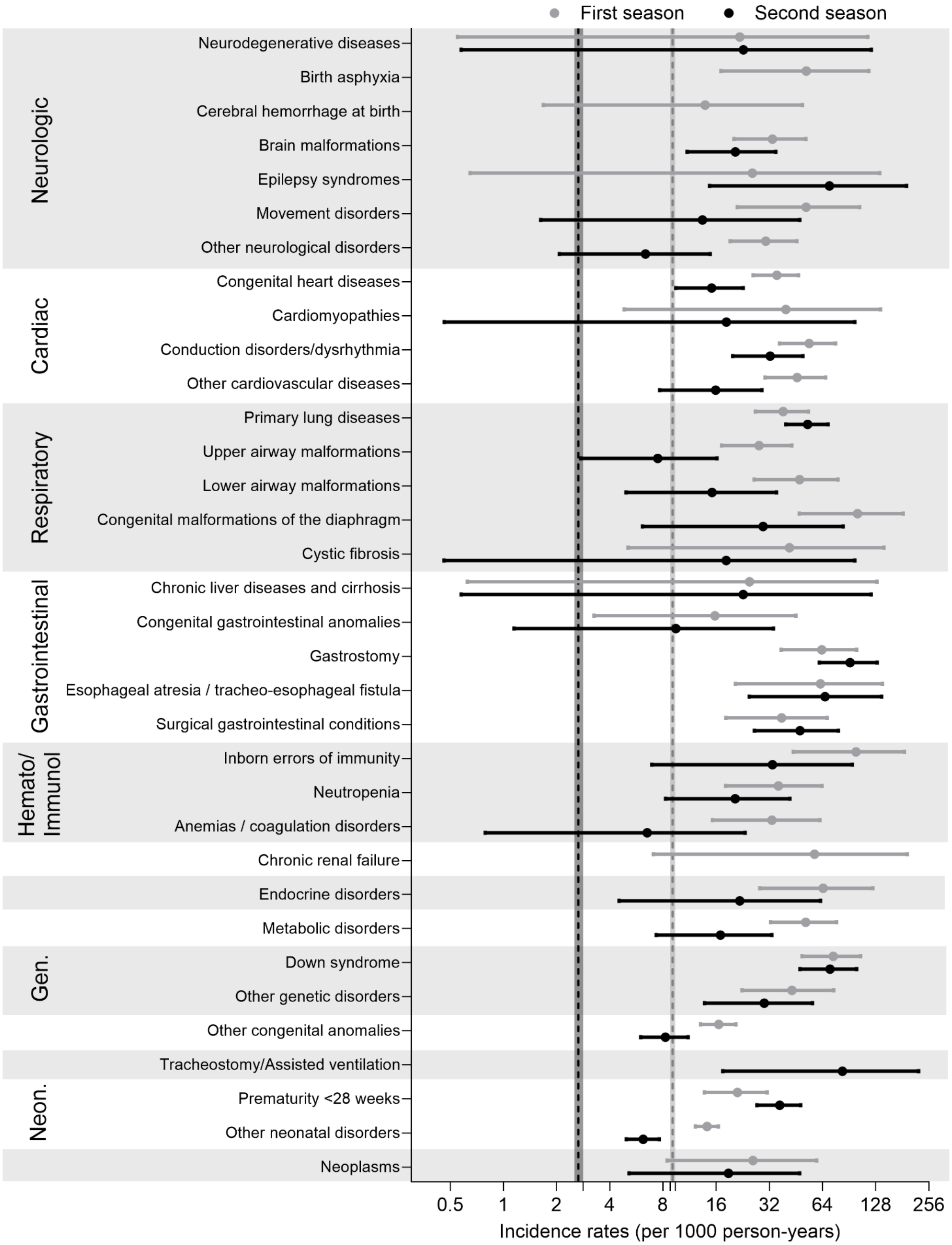

RSV hospitalization incidence rates per 1,000 person-years, with 95% confidence intervals. Vertical lines indicate the rate in all first (light grey) or second (black) season children, with 95% confidence intervals shaded. Children were included if they had at least one ICD-10 code related to disease subgroup (Supplemental Table 1). Some estimates are not shown as there were zero RSV hospitalization events. Hematologic or immunologic (Hemato/Immunol); Genetic (Gen.); Neonatal (Neon.).

© 2025 Viñeta Paramo M et al. *JAMA Network Open.*

eTable 1. List of *ICD-10* Codes Included With Each CMC Subgroup

CATEGORY	SUBCATEGORY	ICD-10 CODES
Neurologic	Neurodegenerative diseases	E75.0, E75.1, E75.2, E75.3, E75.4, E75.5, E75.6, G12.0, G31.8, G31.89, G31.9, G71.0, G71.1, G71.2, G71.3, G71.8, G71.9
	Birth asphyxia	P21.9
	Cerebral hemorrhage at birth	P10.0, P10.1, P52.4, P52.8
	Brain malformation	Q00.0, Q00.1, Q00.2, Q01.0, Q01.1, Q01.2, Q01.8, Q01.9, Q03.0, Q03.1, Q03.8, Q03.9, Q04.X, Q05.X, Q06.X, Q07.0, Q07.8, Q07.9
	Epilepsy syndrome	G40.11, G40.21, G40.301, G40.311, G40.41, G40.804, G40.911, G40.919
	Movement disorders	G24.8, G25.3, G25.81, G25.82, G25.83, G25.89, G25.9
	Other neurological disorders	Q85.0, Q02., G91.1, G93.1, G93.5, G93.8, G94., G95.89, I63.5, P57.0, Q85.1, Z98.2
	Brain tumor	D43.0, D43.1, D43.2, D43.3, D43.4, D43.7, D43.9
Cardiovascular	Congenital heart disease	Q20.0, Q20.1, Q20.2, Q20.30, Q20.31, Q20.32, Q20.38, Q20.4, Q20.50, Q20.58, Q20.6, Q20.8, Q20.9, Q21.2, Q21.3, Q24.X, Q25.1, Q25.3, Q25.4, Q25.5, Q25.6, Q25.7, Q25.8, Q25.9, Q26.X, Q28.2, Q28.3, Q28.9
	Cardiomyopathies	I42.0, I42.1, I42.2, I42.3, I42.4, I42.5, I42.6, I42.7, I42.8, I42.9, I43.0, I43.1, I43.2, I43.8
	Conduction disorder/dysrhythmia	I44.0, I44.1, I44.2, I44.3, I44.4, I44.5, I44.6, I44.7, I45.X, I47.0, I47.1, I47.2, I47.9, I48.0, I48.00, I48.01, I48.02, I48.1, I48.3, I48.4, I48.90, I48.91, I49.00, I49.01, I49.1, I49.3, I49.8, I49.9, I50.9, R00.1, Z45.01, Z95.0
	Other cardiovascular disease	I34.0, I51.81, T82.1, T82.5, T82.7, Z95.2, Z95.81, M30.3
Respiratory	Primary lung disease	I27.0, I27.2, P25.0, P25.8, P27.0, P27.1, P27.8
	Upper airway malformation	Q30.0, Q30.1, Q30.2, Q30.3, Q30.8, Q30.9, Q31.0, Q31.1, Q31.2, Q31.3, Q31.5, Q31.8, Q31.9
	Lower airway malformation	Q32.0, Q32.10, Q32.11, Q32.12, Q32.13, Q32.18, Q32.2, Q32.3, Q32.4, Q33.0, Q33.1, Q33.2, Q33.3, Q33.4, Q33.5, Q33.6, Q33.8, Q33.9, Q34.0, Q34.1, Q34.8, Q34.9
	Congenital hypoventilation syndromes	G47.35
	Cystic fibrosis	E84.0, E84.1, E84.8, E84.9
	Tracheostomy/assisted ventilation	J95.00, J95.01, J95.02, J95.03, J95.04, J95.09, J95.850, Z43.0, Z93.0, Z99.11, Z99.12
Gastrointestinal	Chronic liver disease and cirrhosis	K74.0, K74.1, K74.2, K74.3, K74.4, K74.5, K74.6, K76.8
	Congenital anomalies	Q79.2, Q79.3, Q79.5, Q79.59
	Gastrostomy	Z43.1, Z93.1

© 2025 Viñeta Paramo M et al. *JAMA Network Open.*

	Esophageal atresia +/- tracheoesophageal fistula	Q39.0, Q39.1, Q39.2
	Surgical	Z43.2, Z43.3, Z43.4, Z90.49, Z93.2, Z93.3, Z93.4, Y83.3
	Other gastrointestinal disease	K51.0, K51.1, K51.2, K51.3, K51.4, K51.5, K51.8, K51.9
Hematologic or immunologic	Inborn error of immunity	D84.1, D8.0X, D82.X, D84.0, D84.8, D84.9, D8.1X
	Neutropenia	D70.0
	Hemophagocytic lymphohistiocytosis	D76.1
	Hereditary or aplastic anemia / coagulation disorders	D55.X, D56.X, D57.0, D57.1, D57.2, D57.3, D57.8, D58.X, D60.0, D60.1, D60.8, D60.9, D61.X, D66.X, D68.2, D69.4
	Other immunological disease	B21., B22., B23., B24.
Renal and Urologic	Chronic renal failure	N18.0, N18.1, N18.2, N18.3, N18.4, N18.5, N18.8, N18.9, Z91.15, Z99.2
	Other	N31.9
Endocrine disorders	Endocrine disorders	E22.2, E23.0, E23.2, E25.0, E26.8
Metabolic disorders	Metabolic disorders	E71.0, E71.1, E71.3, E72.1, E72.2, E72.3, E74.0, E74.2, E78.1, E80.6, E80.7, E83.3, E83.4, E88.0, E88.1, E88.8
Congenital / Genetic disorders	Other genetic disorders	Q81.0, Q81.1, Q81.2, Q81.8, Q81.9, Q87.81, Q91.3, Q91.7, Q92.8, Q93.0, Q93.1, Q93.2, Q93.3, Q93.4, Q93.5, Q93.6, Q93.7, Q93.8, Q93.9, Q96.9, Q97.0, Q97.1, Q97.2, Q97.3, Q97.8, Q97.9, Q98.0, Q98.1, Q98.2, Q98.3, Q98.4, Q98.5, Q98.6, Q98.7, Q98.8, Q98.9, Q99.8, Q99.9
	Down syndrome	Q90.9
	Congenital malformation of the diaphragm	K44.9, Q79.0, Q79.1
	Other congenital anomalies	Q41.X, Q42.X, Q43.X, Q44.X, Q45.X, Q60.X, Q61.X, Q62.0, Q62.10, Q62.11, Q62.18, Q62.2, Q62.3, Q62.4, Q62.5, Q62.6, Q62.7, Q62.8, Q63.X, Q64.0, Q64.10, Q64.18, Q64.2, Q64.30, Q64.31, Q64.32, Q64.38, Q64.4, Q64.5, Q64.6, Q64.70, Q64.71, Q64.78, Q64.8, Q64.9, Q75.0, Q75.2, Q75.9, Q76.0, Q76.4, Q76.6, Q76.7, Q77.X, Q78.0, Q78.8, Q78.9, Q79.9, Q87.89, Q89.7, Q89.9
Premature and Neonatal	Extreme prematurity	P07.01, P07.02, P07.21, P07.22, P07.23, P07.24, P07.25, P05.9, P05.2
	Other neonatal disorders	P35.1, P61.3, P61.4, P83.2, P91.2, P91.6
Transplantation	Transplantation	T86.0, T86.4, Z94.4, Z94.81, Z94.82, Z94.83, Z94.84
Neoplasms	Neoplasms	C00.0

The original PCCC list was modified to exclude ICD-10 codes: I51.7 (*Cardiomegaly*), K56.2 (*Volvulus*), P25.1 (*Pneumothorax originating in the perinatal period*), P25.2 (*Pneumomediastinum originating in the perinatal period*), P25.3 (*Pneumopericardium originating in the perinatal period)* and P28.0 (*Primary atelectasis of the newborn*), as these conditions were felt to be unlikely to result in chronic medical conditions.

© 2025 Viñeta Paramo M et al. *JAMA Network Open.*

eTable 2. Data Sources Used for Analysis

DATASET	DATE RANGES FOR AVAILABLE DATA
**British Columbia Centre for Disease Control (BCCDC), Provincial Heath Services Authority (PHSA) and Regional Health Authority data sources:**	
Provincial Laboratory Information Solution (laboratory tests from private/public labs)[1]	Jan 2020-onward
Sunquest Laboratory Information System [2]	Jan 2008-onward
**Ministry of Health (MoH) Administrative Data Sources:**	
Client Roster (CR) (registry of enrolment in the universal public health insurance plan including residential history)[3]	2008/9-onward
Discharge Abstracts Database (DAD) (hospital discharge records)[4]	2008/9-onward
BC Vital Statistics (VS) (deaths registry) [5]	2008/9-onward

Data were obtained through the Provincial Health Service Authority's (PHSA) Platform for Analytics and Data (PANDA), which evolved from a proof-of-concept cloud platform established at the beginning of the COVID-19 pandemic(13). The integrated PANDA platform provides data, governance, tools and services to support reporting and analytics for users and researchers across PHSA.

[1] Provincial Health Services Authority [creator]. Provincial Public Health Information Systems [publisher]. (2020). 2021.

[2] Provincial Health Services Authority (PHSA), Vancouver Coastal Health (VCH) and Providence Health Care (PHC) [creator]. Sunquest Laboratory Information System. Provincial Laboratory Medicine Services [publisher]. Data Extract. PLMS (2020). 2024.

[3] British Columbia Ministry of Health [creator]. Client Roster (Client Registry System/Enterprise Master Patient Index). British Columbia Ministry of Health [publisher]. Data Extract. MOH (2020). 2024. https://www2.gov.bc.ca/gov/content/health/health-forms/online-services

[4] British Columbia Ministry of Health [creator]. Discharge Abstract Database (Hospital Separations). British Columbia Ministry of Health [publisher]. Data Extract. MOH (2020). 2021. https://www2.gov.bc.ca/gov/content/health/health-forms/online-services

[5] BC Vital Statistics Agency [creator]. Vital Statistics Deaths. BC Vital Statistics Agency [publisher]. Data Extract. BC Vital Statistics Agency. 2021. https://www2.gov.bc.ca/gov/content/health/health-forms/online-services

© 2025 Viñeta Paramo M et al. *JAMA Network Open.*

eTable 3. RSV Hospitalizations Outcomes Between First-Season Children With or Without Chronic Medical Conditions (CMCs), by Season

	First RSV season	
	CMCs<br>N = 25,452	No CMCs<br>N = 406,485
Hospitalized for RSV-LRI during that season, n (% children)	382 (1.5)	3,045 (0.8)
One RSV-LRI episode, n (% hospitalized)	378 (99.0)	3,033 (99.6)
Two RSV-LRI episodes, n (% hospitalized)	<6	12 (0.4)
In-season RSV hospitalizations for RSV-LRI, n children	341	2,781
Age at first RSV-LRI episode in months, median (Q1, Q3)	3.5 (1.6, 6.4)	2.2 (1.2, 4.5)
RSV hospitalization in previous season, n (% hospitalized)	-	-
Length of hospital stay in days, median (Q1, Q3)	4 (2, 7)*	3 (2, 5)
PICU admission, n (% hospitalized)	76 (19.7)*	337 (11.0)
Length of PICU admission in days, median (Q1, Q3)	5 (3, 8)*	4 (2, 5)
Mechanical ventilation, n (% hospitalized)	28 (7.3)*	107 (3.5)
Unadjusted hospitalization IRs per 1,000 PY	17.75 (16.05 – 19.59)	8.55 (8.25 – 8.86)
Adjusted[£] RSV hospitalization IRs, per 1,000 PY	15.85 (14.16 – 17.55)	8.02 (7.74 – 8.31)
Adjusted[¥] RSV hospitalization IRs, per 1,000 PY	13.59 (12.09 – 15.10)	8.13 (7.84 – 8.42)

Adjusted RSV hospitalization incidence rates (IRs) per 1,000 person-years, with 95% confidence intervals, estimated using a Poisson Generalized Estimating Equation model with an exchangeable correlation structure, and an offset term for [£]follow-up time, sex and administration of palivizumab during the same season or [¥] follow-up time, sex, administration of palivizumab during the same season and prematurity.

[*]Exposure groups defined by presence of at least one chronic medical condition (CMCs).

In-season refers to events occurring between October 1st and March 31st.

Pediatric intensive care unit (PICU) admission defined as admissions involving the intensive care unit at BC Children's Hospital and Victoria General Hospital. Mechanical ventilation based on the Canadian Institute of Health Information intervention codes: 1.GZ.31.CA-ND and 1.GZ.31.CR-ND.

*P-value<0.001 comparing the CMCs vs. no CMCs children's groups in the first season, using a Mann-Whitney-Wilcoxon test for length of hospital and PICU stay and with Chi-square for proportion of PICU admission and need for mechanical ventilation.

© 2025 Viñeta Paramo M et al. *JAMA Network Open.*

eTable 4. Sensitivity Analysis With Modified Main Outcome Definitions

	**RESTRICTED TO PRE-PANDEMIC**		
	First season	Second season	
	All children	CMCs	No CMCs
Total children	219,420	12,573	206,847
RSV hospitalizations	1,833	110	443
Unadjusted hospitalization IR per 1,000 PY	9.52 (9.09 – 9.97)	9.16 (7.57 – 11.00)	2.15 (1.96 – 2.36)
Adjusted[£] RSV hospitalization IR per 1,000 PY	8.88 (8.47 – 9.28)	8.47 (6.87 – 10.08)	2.03 (1.84 – 2.22)
Adjusted[£] absolute risk difference between groups per 1,000 PY	Ref.	-0.40 (-2.05 – 1.25)	-6.85 (-7.29 – -6.40)
Adjusted[£] incidence rate ratios between groups	Ref.	0.95 (0.79 – 1.16)	0.23 (0.21 – 0.25)
	**EXPANDED OUTCOME DEFINITION***		
	First season	Second season	
	All children	CMCs	No CMCs
Total children	431,937	24,723	406,116
RSV hospitalizations	3,727	230	1,101
Unadjusted hospitalization IR per 1,000 PY	9.82 (9.51 – 10.15)	9.34 (8.19 – 10.60)	2.71 (2.56 – 2.88)
Adjusted[£] RSV hospitalization IR per 1,000 PY	9.17 (8.88 – 9.47)	8.69 (7.56 – 9.82)	2.56 (2.41 – 2.72)
Adjusted[£] absolute risk difference between groups per 1,000 PY	Ref.	-0.48 (-1.65 – -0.68)	-6.61 (-6.94 – -6.28)
Adjusted[£] incidence rate ratios between groups	Ref.	0.95 (0.83 – 1.08)	0.28 (0.26 – 0.30)

Adjusted RSV hospitalization incidence rates (IRs) per 1,000 person-years, with 95% confidence intervals, estimated using a Poisson Generalized Estimating Equation model with an exchangeable correlation structure, and an offset term for [£]follow-up time, sex and administration of palivizumab during the same season

* Expanded outcome definition includes the primary outcome (hospitalizations with RSV-LRI ICD-10 codes) and any hospitalization with a most responsible diagnosis due to respiratory symptoms (ICD-10 starting with "J") and a positive RSV test within 7 days of admission.

© 2025 Viñeta Paramo M et al. *JAMA Network Open.*

eMethods. Primary Outcome Validation (RSV Hospitalizations)

The *primary outcome* was hospital admission with most responsible or secondary diagnosis of RSV-related LRI (RSV-LRI), based on the following ICD-10 codes: J12.1 (*RSV pneumonia*), J20.5 (*Acute bronchitis due to RSV*), J21.0 (*Acute bronchiolitis due to RSVI),* B97.4 (*Respiratory syncytial virus as the cause of diseases classified elsewhere*). This document describes the validation of this outcome using ICD-10 codes for identification of RSV-related hospitalizations due to lower respiratory infection (RSV-LRI). The validation analysis had two main goals: (1) examine the variability of RSV-LRI ICD-10 codes regional Health Authorities, annual seasonal periods and age groups, and (2) determine the accuracy of using RSV-LRI ICD-10 codes as the study's main outcome. Of note, no ICD-9 codes were captured, as the data source utilizes only ICD-10 for the study period.

Methodology: First, *respiratory* ICD-10 codes (i.e. those starting with "J") were grouped into seven categories according to eTable 5. Data from children hospitalized with these codes as the admission's associated "most responsible diagnosis" were then linked to respiratory testing data, as the "source of truth", if testing occurred ±7 days of hospital admission.

When multiple tests were conducted within this 7-day period, any positive result was considered, and multiple tests with the same results were merged into one entry, keeping the earliest date.

eTable 5 shows that the combination of ICD-10 codes for RSV-LRI represented the highest percentage of children with available respiratory testing data (63.1% of RSV-LRI episodes). This category was also the one with the highest testing positivity rate (PR; 96.4% of all respiratory tests were positive for RSV). This notably differentiates from the following category with the highest positivity rate (23% for category *Pneumonia*).

Second, testing results for each of the seven respiratory categories were evaluated for differences between geographical location (i.e. by British Columbia Health Authority), annual season of RSV diagnosis (September 1st to August 31st of the following year), or age strata (eTable 5-7). The results showed that the RSV-LRI category had the greatest proportion of positive RSV tests among all the strata. Of note, the proportion of children with no testing data available varied between Health Authorities (eFigure 10). The proportion of children with no testing available also varied between annual seasonal periods, with the lowest number of respiratory testing done during the 2020-2021 pandemic year (eFigure 11). Also, the RSV-LRI category predominated under six months, but remained the one with the most positive RSV tests among all age groups.

Among children who had respiratory testing, the sensitivity and specificity of the most responsible diagnosis of RSV-LRI at the time of hospital admission, based on ICD-10 codes, was determined, comparing with RSV testing data as the source of truth. This definition showed 98.0% specificity and 78.2% sensitivity (eTable 6).

Respiratory admissions not classified as RSV-LRI (according to most responsible diagnosis) included some children with positive RSV testing results (eTable 7, eFigure 13). Children with a secondary diagnosis of RSV-LRI by ICD-10 code had a positive RSV test in 69.1% of cases (i.e. 94 children tested positive out of 136 who had RSV-LRI as a secondary diagnosis during a hospital admission). Conversely, most children with available testing data that did not have an RSV-LRI secondary diagnosis based on ICD-10 codes tested negative for RSV (total N=5,157 tested negative; N=710 tested positive; N=4,447 tested negative, for RSV); eTable 7). Of the children who had RSV-LRI as a secondary diagnosis, 46.9% did not have available testing results. Once the children with RSV-LRI by ICD-10 code as most responsible secondary diagnosis were included, the specificity was 97.9%, and the sensitivity was 80.7%, compared with the RSV testing gold standard (eTable 7). Therefore, children for whom RSV-LRI ICD-10 codes were considered as a secondary diagnosis were also included in the main outcome for RSV-related hospitalizations.

© 2025 Viñeta Paramo M et al. *JAMA Network Open.*

Interpretation: The use of RSV-LRI as most responsible or *secondary* diagnoses, based on ICD-10 codes, showed 97.9% specificity and 80.7% sensitivity in identifying hospitalized children with RSV infections, based on positive respiratory testing, among hospitalized children. Therefore, this outcome was used as primary outcome for the current study. While the specificity of this definition was high when only considering RVS-LRIs as primary diagnosis, adding children with an RSV-LRI as secondary diagnosis increased sensitivity from 78.1% to 80.7%.

The data source had a relatively large proportion of children who did not get tested (43.7% of the children hospitalized for respiratory symptoms do not have testing data linked), justifying the choice of ICD-10 codes for case identification, rather than directly using respiratory testing data (the gold standard). The lack of RSV testing data hospitalized children may have been due to provider practices or inconsistent reporting of testing data between Health Authorities and across annual periods in the source database used for this study.

A relatively small minor proportion of children diagnosed with an RSV-LRI had a negative RSV testing result, suggesting a false positive rate of 2.1% for the main RSV-LRI outcome. Some individuals who had a positive RSV testing result required multiple hospital admissions and thus re-testing within 7 days may have resulted in a negative test result. In other cases, the explanation could simply be that the ICD-10 was miscoded.

Conclusion: The main study outcome retained for analyses was ANY admissions within the first two years of age where the most responsible (primary) or secondary diagnosis was a RSV-LRI, using the following the ICD-10 codes: J12.1 (RSV pneumonia), J20.5 (Acute bronchitis due to RSV), J21.0 (Acute bronchiolitis due to RSV). No children were hospitalized with the B97.4 ICD-10 code.). No hospitalized children in the cohort were identified with the B97.4 ICD-10 code (Respiratory syncytial virus as the cause of diseases classified elsewhere).

Additionally, to address a possible measurement bias due to ICD-10 miscoding, a sensitivity analysis was conducted adding to the primary outcome any hospitalization with respiratory symptoms as most responsible diagnosis (ICD-10 codes starting with “J”) AND positive RSV test within 7 days of admission.

© 2025 Viñeta Paramo M et al. *JAMA Network Open.*

eMethods figures and tables:

eTable 5. Proportions of Hospitalized Children in Cohort, With Positive RSV Tests by Respiratory Diagnosis Categories Based on *ICD-10* Codes

Diagnosis categories	ICD-10 codes	Total children	% with test available	Positive tests (% of tests)	Negative tests (% of tests)	Testing not available
RSV-LRI	J20.5, J12.1, J21.0, B97.4	4,740	63.1	2886 (96.4)	107 (3.6)	1,747
Pneumonia (bacterial or unspecified etiology)	J15.X, J18.X	1,277	50.7	149 (23.0)	499 (77.0)	629
LRI with unspecified etiology	J12.9, J18.0, J20.9, J21.9, J22.X, J21.8	4,263	57.2	376 (15.4)	2,061 (84.6)	1,826
Asthma	J45.X	1,161	55.2	81 (12.6)	560 (87.4)	520
Other	All other JX.X codes	1,294	50.4	60 (9.2)	592 (90.8)	642
URI	J0X.X	2,755	48.3	121 (9.1)	1,210 (90.9)	1,424
Influenza RI	J10.X, J11.X	433	59.6	17 (6.6)	241 (93.4)	175
Totals		15,923	56.3	3,681	5,270	6,963

This table includes all respiratory hospital admissions based on ICD-10 codes starting with “J”, reported as “most responsible diagnosis” within 2 years of age. in the cohort. LRI: lower respiratory tract infection, URI: upper respiratory infection, RI: respiratory infection.

© 2025 Viñeta Paramo M et al. *JAMA Network Open.*

eTable 6. Sensitivity and Specificity (95% Confidence Interval in Brackets) With of Using RSV-LRI as a Most Responsible Primary Diagnosis Among Hospitalized Children, Compared to RSV Testing Results

		**RSV-LRI ICD-10 as most responsible diagnosis**		**RSV-LRI ICD-10 as most responsible diagnosis OR as secondary diagnosis**	
		YES	NO	YES	NO
**Ref**	**RSV testing POSITIVE**	2,886	804	2,980	710
	**RSV testing NEGATIVE**	107	5163	113	5,157
		Sensitivity: 78.2% (76.8 – 79.5)<br>Specificity: 98.0% (97.4 – 98.2)<br>Positive predictive value:<br>96.4% (95.4 – 96.8)<br>Negative predictive value:<br>86.5% (85.6 – 87.4)		Sensitivity: 80.7% (79.4 – 82.0)<br>Specificity: 97.9% (97.2 – 98.1)<br>Positive predictive value:<br>96.1% (95.3 – 96.7)<br>Negative predictive value:<br>87.9% (87.0 – 88.7)	

True negatives = no RSV-LRI and negative RSV testing.

© 2025 Viñeta Paramo M et al. *JAMA Network Open.*

eTable 7. RSV Respiratory Testing Results in Children With a Most Responsible Diagnosis Other Than RSV-LRI, Stratified According to the Presence of RSV-LRI as a Secondary Admission Diagnosis

	**RSV-LRI ICD-10 as secondary diagnosis**	
	**YES, n (%)**	**NO, n (%)**
**RSV testing POSITIVE**	94 (69.1)	710 (6.4)
**RSV testing NEGATIVE**	6 (4.4)	5,157 (46.7)
**RSV testing not available**	36 (26.5)	5,180 (46.9)

© 2025 Viñeta Paramo M et al. *JAMA Network Open.*

eFigure 10. RSV Testing Results in Children Requiring Hospital Admissions With a Most Responsible Diagnosis in the Respiratory Category (JXX.X), by De-Identified Health Authorities in British Columbia (A, B, …)

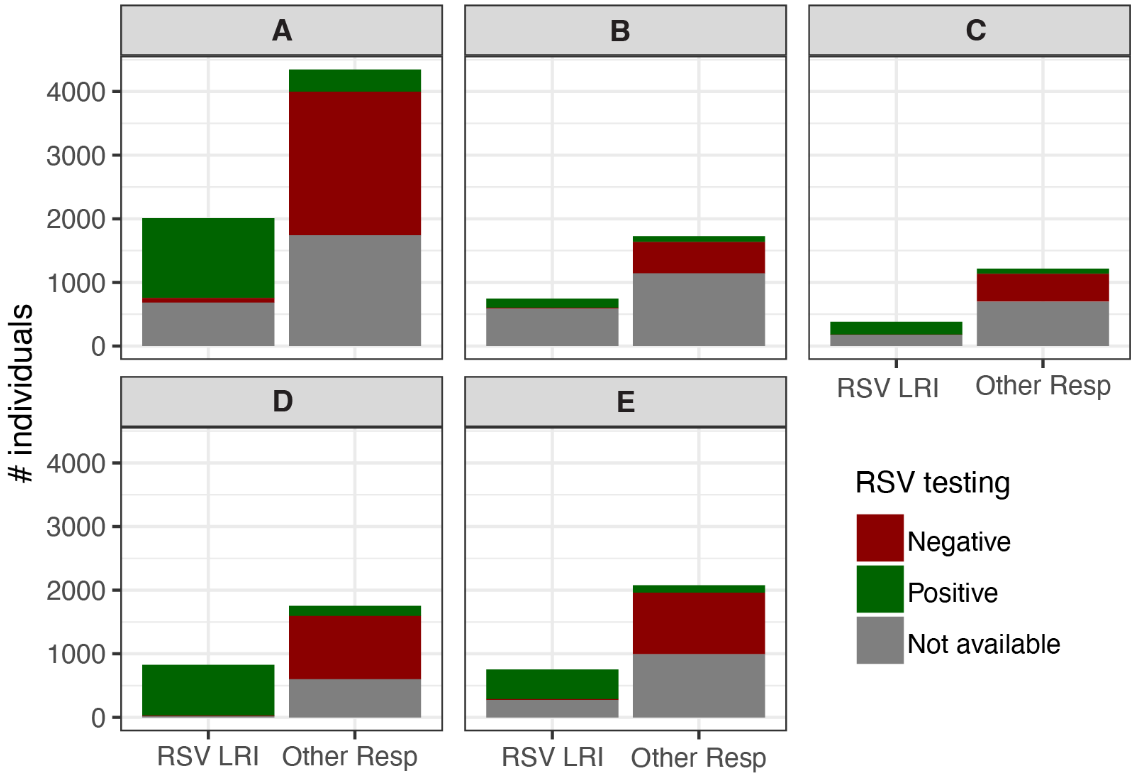

RSV-LRI defined based on ICD-10 codes: J20.5, J12.1, J21.0, B97.4, and all other categories defined in **eTable 5** were combined as “Other Resp.”. The “not available” category may include individuals who were not tested or who were tested but for whom data was not reported to the data source for this project.

© 2025 Viñeta Paramo M et al. *JAMA Network Open.*

eFigure 11. RSV Testing Results in Children Requiring Hospital Admissions With a Most Responsible Diagnosis in the Respiratory Category (JXX.X), by Annual Season Period (September 1 to August 31 of the Following Year)

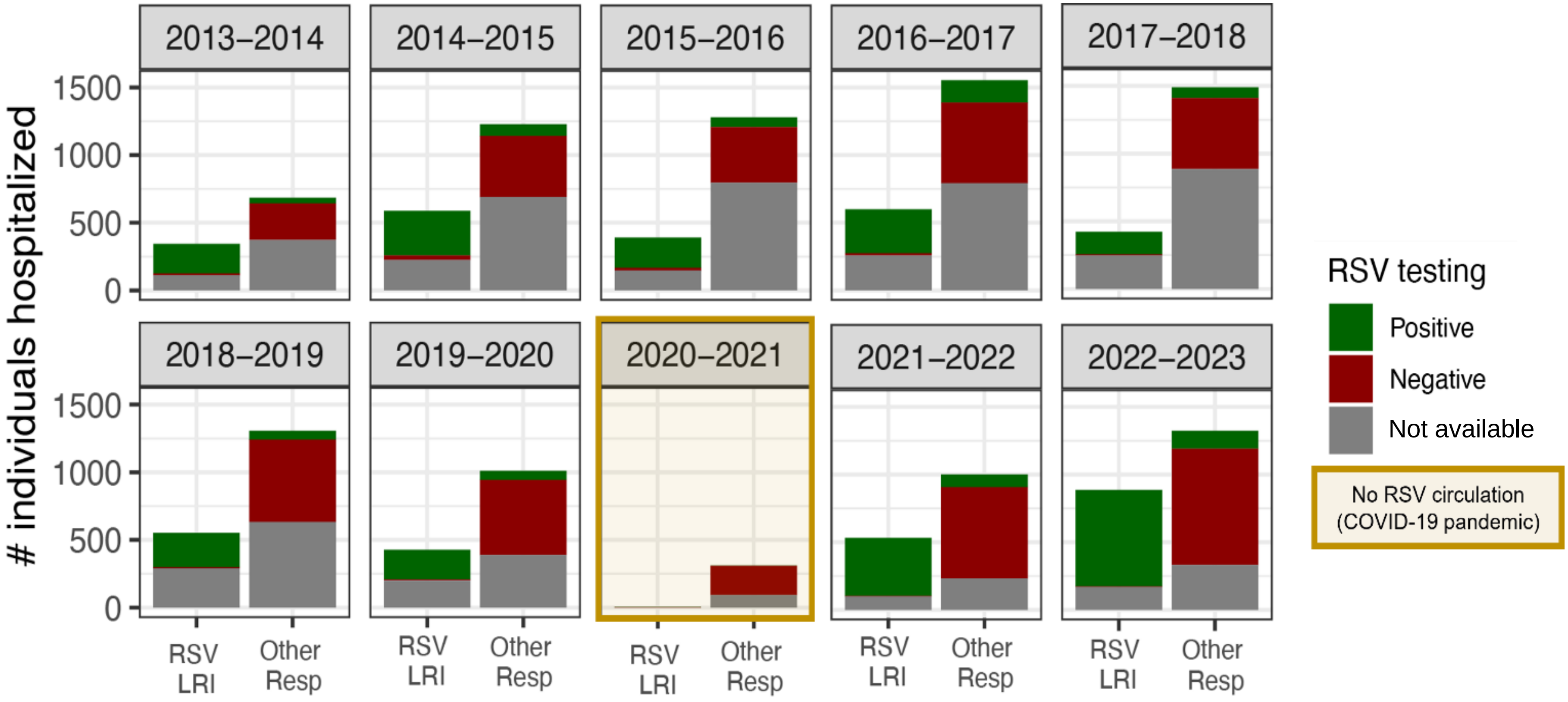

Categories shown are RSV-LRI, based on ICD-10 codes: J20.5, J12.1, J21.0, B97.4, and all other respiratory diagnosis categories combined as "other resp.". The "not available" group includes children who were not tested or who were tested but for whom data was not reported to the data source for this project. The 2020-2021 annual period was atypical in British Columbia with only a few cases reported throughout entire period. Most RSV tests were conducted using multiplex PCR. However, during the earlier years of the study period (2013–2016), a minority of tests may have been performed using direct fluorescent antibody (DFA) assays (unpublished data).

© 2025 Viñeta Paramo M et al. *JAMA Network Open.*

eFigure 12. RSV Testing Results in Children Requiring Hospital Admissions With a Most Responsible Diagnosis in the Respiratory Category (JXX.X), by Age Groups

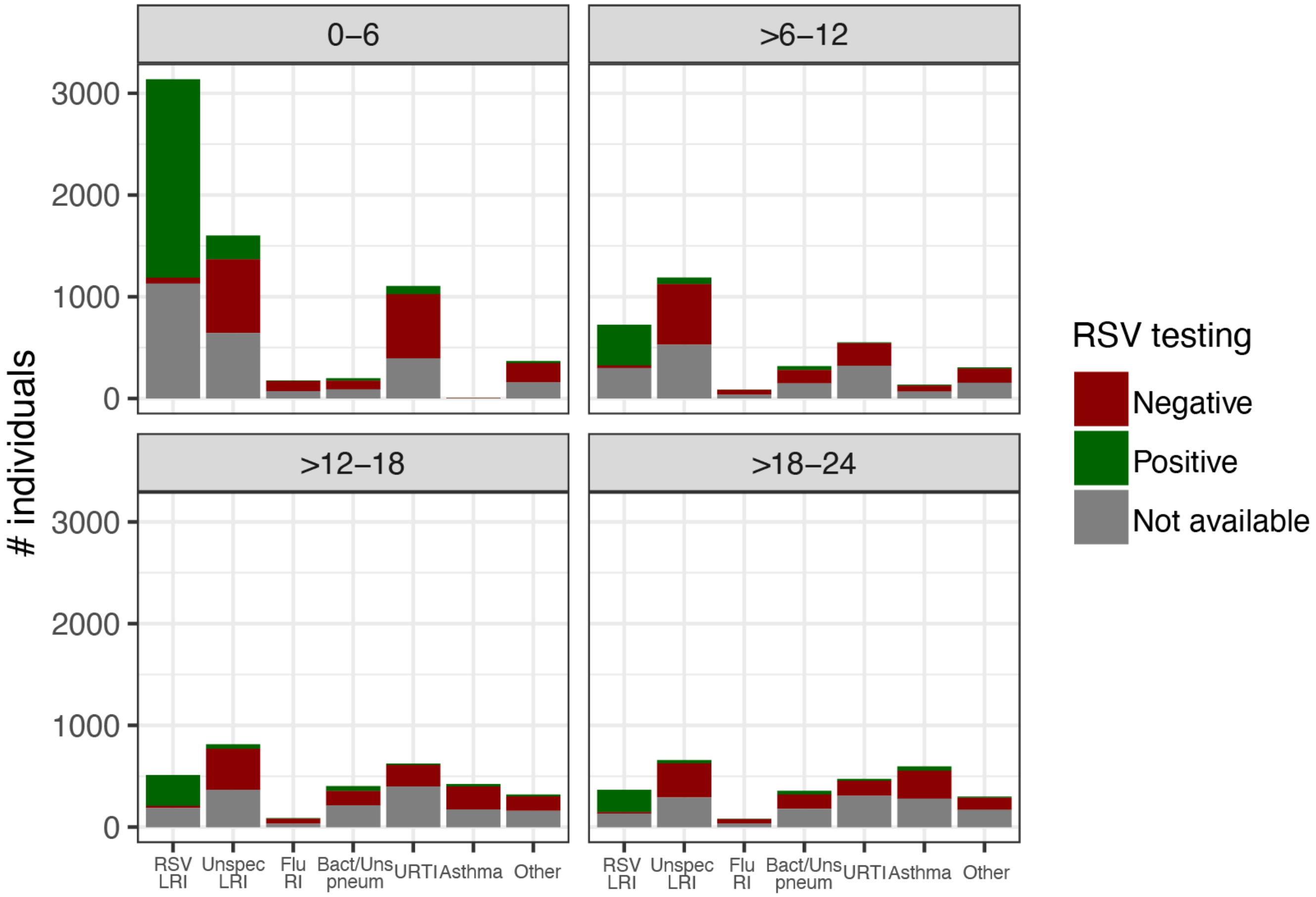

Categories shown are RSV-LRI, based on ICD-10 codes: J20.5, J12.1, J21.0, B97.4, and all other respiratory diagnosis categories combined as “other resp.”. The “not available” group includes children who were not tested or who were tested but for whom data was not reported to the data source for this project. LRI: lower respiratory tract infection, URTI: upper respiratory tract infection, RI: respiratory infection.

© 2025 Viñeta Paramo M et al. *JAMA Network Open.*

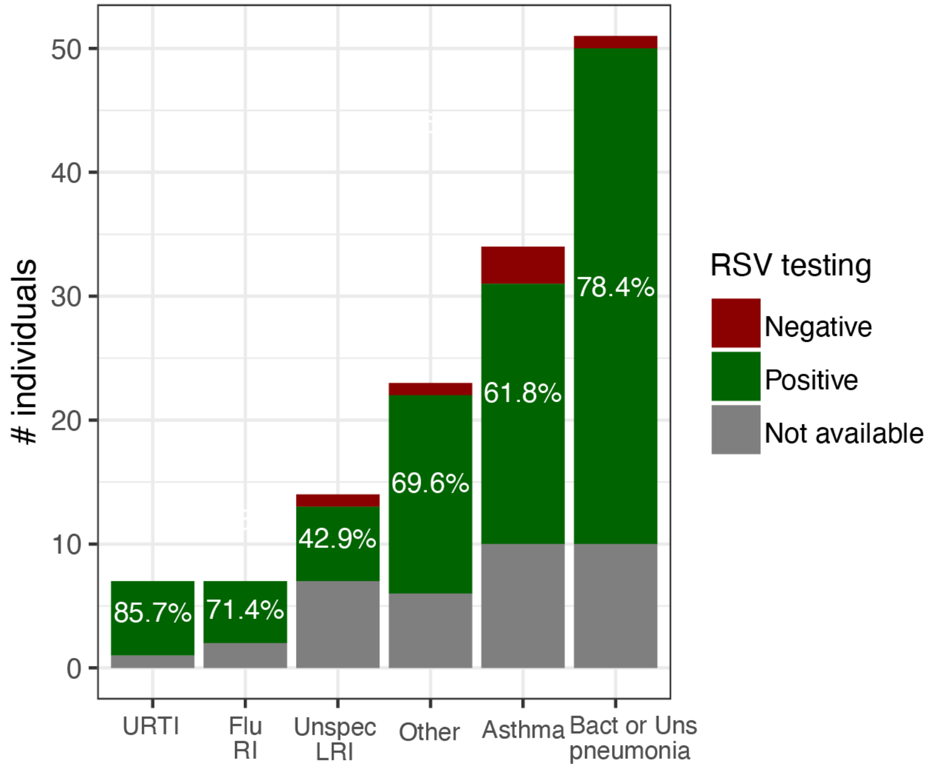

eFigure 13. RSV Testing Results in Children With a Most Responsible Diagnosis Other Than RSV-LRI and RSV-LRI as a Secondary Admission Diagnosis. Percentages represent the proportion of RSV positive test among that category. The “not available” group includes children who were not tested or who were tested but for whom data was not reported to the data source for this project.

© 2025 Viñeta Paramo M et al. *JAMA Network Open.*
