# Supplementary material for: RSV Hospital Admissions During the First 2 Seasons Among Children With Chronic Medical Conditions
Source: JAMA Netw Open. 2025 Jul 8;8(7):e2519410. doi: 10.1001/jamanetworkopen.2025.19410 (PMC12238895; doi:10.1001/jamanetworkopen.2025.19410)
Supplement: Supplement 3. — Data Sharing Statement [file jamanetwopen-e2519410-s003.pdf]

Data Sharing Statement

Viñeta Paramo. RSV Hospital Admissions During the First 2 Seasons Among Children With Chronic Medical Conditions. *JAMA Netw Open*. Published July 08, 2025. doi:10.1001/jamanetworkopen.2025.19410

Data
Data available: No

Additional Information
Explanation for why data not available: The data used in this study come from provincial health administrative data. The authors will share the data dictionary upon request. Actual data requests should be made to Population Data BC through their portal (www.popdata.bc.ca).
